# Five Fungal Pathogens Are Responsible for Bayberry Twig Blight and Fungicides Were Screened for Disease Control

**DOI:** 10.3390/microorganisms8050689

**Published:** 2020-05-08

**Authors:** Wenjun Li, Ming Hu, Yang Xue, Zhijun Li, Yanfei Zhang, Daoxu Zheng, Guangtao Lu, Junxia Wang, Jianuan Zhou

**Affiliations:** 1 Guangdong Laboratory for Lingnan Modern Agriculture, Guangdong Province Key Laboratory of Microbial Signals and Disease Control, Integrative Microbiology Research Center, South China Agricultural University, Guangzhou 510642, China; lookitso@hotmail.com (W.L.); hm13@stu.scau.edu.cn (M.H.); 18819455948@163.com (Y.X.); hnnydxlzj@163.com (Z.L.); aquamarineeee@126.com (Y.Z.); 2 Shantou Forestry Research Institute, Shantou 515041, China; gdstzdx@163.com; 3 State Key Laboratory for Conservation and Utilization of Subtropical Agro-bioresources, College of Life Science and Technology, Guangxi University, Nanning 530004, China; lugt@gxu.edu.cn

**Keywords:** twig blight disease, *Myrica rubra*, fungicide, *Epicoccum sorghinum*, *Neofusicoccum parvum*, *Lasiodiplodia theobromae*, *Nigrospora oryzae*, *Pestalotiopsis myricae*

## Abstract

Bayberry (*Myrica rubra*) is a commercial fruit in China. For the past seven years, twig blight disease has been attacking bayberry plantations in Shantou City, Guangdong Province, China, leading to destructive damage and financial loss. In this study, five fungal species associated with twig dieback and stem blight were identified based on morphological characteristics combined with multilocus sequence analysis (MLSA) on the internal transcribed spacer (ITS) region, partial sequences of β-tubulin (*tub2*), translation elongation factor 1-α (*tef1*-α), large subunit ribosomal RNA (*LSU*) and small subunit ribosomal RNA (*SSU*) genes, which are *Epicoccum sorghinum*, *Neofusicoccum parvum*, *Lasiodiplodia theobromae*, *Nigrospora oryzae* and a *Pestalotiopsis* new species *P. myricae*. *P. myricae* is the chief pathogen in fields, based on its high isolation rate and fast disease progression after inoculation. To our knowledge, this is the first study reporting the above five fungi as the pathogens responsible for bayberry twig blight. Indoor screening of fungicides indicates that Prochloraz (copper salt) is the most promising fungicide for field application, followed by Pyraclostrobin, 15% Difenoconazole + 15% Propiconazole, Difenoconazole and Myclobutanil. Additionally, *Bacillus velezensis* strain 3–10 and zeamines from *Dickeya zeae* strain EC1 could be used as potential ecofriendly alternatives to control the disease.

## 1. Introduction

Bayberry (*Myrica rubra*), belongs to the genus *Myrica*, *Myricaceae*, Magnoliopsida, and is a subtropical fruit tree grown for its high medicinal and edible value. Presently, commercial plantations of bayberry are mainly distributed in China, Japan, South Korea, India, Myanmar, Vietnam, the Philippines and other South Asian countries [1]. In China, it is planted in the south, including Hunan, Anhui, Zhejiang, Jiangsu, Fujian, Guangdong, Guangxi, Jiangxi, Guizhou, Sichuan, Yunnan and Taiwan Provinces nowadays [1,2]. It originated from Yuyao in Zhejiang Province, where bayberry pollens were discovered during a Neolithic Hemudu site excavation in 1973, suggesting its existence in this area more than 7000 years ago. It is also considered as a traditional garden tree species in classical East Asian gardens.

However, bayberry twig blight disease (also known as bayberry branch and leaf wilt, bayberry canker, or bayberry yellow blight) has been found on bayberry trees grown in South China, but not the rest of the world, leading to destructive damage and financial loss for the past decades. This disease was first spotted on Dongkui cultivar in Huangyan District, Taizhou City, Zhejiang Province, in 1999 [3], and gradually spread to Biqi cultivar in Xianju County in 2004, Dongkui cultivar in Jiaojiang District in 2008 as well as Tiantai and Linhai Counties in 2009 [4], covering an area of about 6163 ha by 2011 [3]. It then spread quickly to the Fujian (Ding’ao cultivar) and Guangdong Provinces (multiple cultivars) in 2013 [5]. The origin of the bayberry cultivars found in Guangdong plantations (Dongkui, Yingsi and Fujian Dahong) was mainly from Zhejiang and Fujian and they are more prone to twig blight disease (almost 100% incidence), whereas those local cultivars from other areas, such as Tuzhong (64.7% incidence), Qingdi (86.6% incidence) and Wusu (95.4% incidence), showed more resistance to the disease [5]. However, local bayberry industry has witnessed a consistently upward tendency of morbidity, and some areas in Shantou City have also suffered from destructive twig blight disease for the past several years.

The cause of bayberry twig blight is very confusing at present. *Pestalotiopsis* spp. were identified as the causal agents of the disease in Zhejiang Province [6], in which, *P. mangiferae* and *P. vismiae* isolated from diseased twigs were first determined to be the pathogens of twig dieback on different bayberry cultivars collected from Huangyan, Xianju, Linhai, Wenzhou, Rui’an and Yueqing in Zhejiang Province and Fu’an in Fujian Province [3,7]. Meanwhile, *P. versicolor* and *P. microspora* (previously claimed as *P. vismiae*) obtained from blighted twig samples were verified as the primary pathogens of bayberry twig blight on Dongkui and Biqi cultivars in Xianju, Rui’an and Huangyan in Zhejiang Province [2]. Furthermore, *P. clavispora* was also isolated from diseased bayberry (Dongkui and Biqi cultivars) leaves showing the symptom of brown leaf spot and determined as the pathogen of bayberry brown spot disease in Zhejiang Province [8]. *Botryosphaeria dothidea* was also frequently isolated from symptomatic root tissues and considered as the causal agent of bayberry sudden dry blight [4,9], which was also previously identified as bayberry root rot [10]. Some researchers considered that improper fertilization method (long-term, single and continuous application of compound fertilizer) or unreasonable compound fertilizer formula are the main causes of the disease, aggravating pathogen infection [10,11].

Recently, we investigated the incidence of bayberry twig blight in Shantou City, Guangdong Province and found that it has become a serious threat to the development of bayberry, covering an area of about 3.73 Km^2^ with 84% of average incidence (data unpublished). We gathered from the farmers that fungicides such as prochloraz, propiconazole, difenoconazole, carbendazim and thiophanate, rather than biocontrol agents, are usually applied to control the disease, but no obvious effect is obtained to prevent and treat the disease. Pathogen identification in our study revealed that infection by at least five pathogenic fungi is the principal cause for the aggressiveness and rapid progression of the disease in this area, especially in humid and hot weather after typhoon transits. This study aims to identify and characterize the pathogens responsible for bayberry twig blight disease and to establish an effective disease control method using a combination of low toxic fungicide compounds and biocontrol bacteria. Furthermore, bacteriocins such as carotovoricin and carocin S1 produced by *Pectobacterium carotovorum* subsp. *carotovorum* (*Pcc*) are potentially biological control agents [12], so we screened some known phytotoxins for developing promising fungicides to control bayberry twig blight. In our previous study, we identified polyamino-amide zeamines from the rice foot rot pathogen *Dickeya zeae* EC1 [13], which showed promising potential as fungicides to control *Peronophythora litchi* [14,15]. This study also tested the inhibitory activity of zeamines on the identified fungal pathogens.

## 2. Materials and Methods

### 2.1. Disease Investigation and Symptomatic Sample Collection

We investigated bayberry twig blight disease in Jinzao town, Shantou City, Guangdong Province, China, from 5th to 11th July, 2018, the location of which is 23°23’55.9" to 23°30’57.9" North latitude and 116°18’44.7" to 116°26’13.7" East longitude, in a southern subtropical monsoon climate with red loam suitable for fruit tree planting. The planting area of the fruit forest in Jinzao town is about 50 Km^2^, in which, bayberry is grown over 10 Km^2^, with mixed planting of local and exotic cultivars. In general, the planting ratio of local cultivars to exotic cultivars is 8:2, which between the local cultivars is Qingdi:Wusu:Kuaimei:Aodi wusu:Shanmei:others (such as Baimei, Tumei, and Baiyesu) = 5:1:1:0.5:0.5:2, and between the exotic cultivars is Dongkui:Fujian Dahong:Yingsi:others = 4:3:2:1.

The bayberry fruits were harvested between early to mid-June when the weather is usually hot and rainy (the temperature was between 25–33 ℃ with frequent thunderstorms at that time), after typhoon days. About 3.73 Km^2^ of bayberry plantations were investigated in Jinzao town and almost all the plantations were seriously caught in twig blight disease, where approximately 33,000 bayberry trees were planted per Km^2^ with other fruit trees interplanted. To study the incidence rate, the Five Sampling Method was used. Briefly, 5 points in each orchard and 12 trees at each point were examined. A total of 600 trees in ten orchards were investigated. For pathogen isolation, diseased branches from a new orchard (Limin Orchard, 4 years old) and an old orchard (Caisong Peng Orchard, 12 years old) were separately collected for moisturizing culture and pathogen isolation in the laboratory.

### 2.2. Isolation and Purification of Pathogens

Symptomatic twigs and leaves were collected from 4 trees (4 years old) from Limin Orchard and 3 trees (12 years old) from Caisong Peng Orchard in Jinzao town bayberry plantations. Diseased samples were washed with sterile water and cut into small pieces (0.5–1 cm^2^), then surface-sterilized with 70% ethanol solution for 30 s and 5% sodium hypochlorite for 2 min, before washing with sterile water three times. Tissues were placed on sterile filter paper to air dry and then transferred onto potato dextrose agar (PDA, TOPBIO, Zhaoyuan, China) plates for incubation at 28 °C for 3 days. When mycelia developed from the tissues, they were isolated and purified onto fresh PDA plates using a single spore or hyphal tip separating technique, after which they were transferred to PDA slants and kept at 4 °C for further studies.

### 2.3. Morphological Characterization

Pure isolates were cultured on PDA, malt extract agar (MEA, TOPBIO, Zhaoyuan, China), oat agar (OA, TOPBIO, China) and water agar (0.8%) (WA) plates for 7 days at 28 °C. The morphology of the fungal colonies was captured by a Nikon D750 (Nikon, Ayutthaya, Thailand). Different methods were used to induce sporulation for the isolates. After 7 days of culture, conidia were generated on PDA plates for isolate P4, on sterile bayberry twigs on WA plates for isolate E1, and induced by ultraviolet radiation on bayberry twigs on WA plates for isolates L3 and N9, while isolate F7 could not sporulate successfully on PDA, MEA, OA or WA media with or without ultraviolet induction. Fungal mycelia and spores were observed and captured by a Zeiss Axio observer Z1 Microscope (Carl Zeiss Microscopy, Jena, Thueringen, Germany). The sizes of 30 conidia were measured for each sporogenic isolate. All microscopic measurements were recorded with ZEM PRO 2012 software (Carl Zeiss Microscopy, Germany).

### 2.4. Multilocus Sequence Analysis (MLSA) and Phylogenetic Analysis

Mycelia from fresh cultures were scraped from the surface of the PDA plates incubated at 25 °C for 3 to 5 days. Total genomic DNA of each isolate was extracted by Omega E.Z.N.A. Fungal DNA Miniprep Kit. The *ITS*, *tub2*, *tef1-α*, *LSU* and *SSU* sequences were respectively amplified using the primer pairs listed in Table S1 [16,17,18,19]. PCR was performed in a BIORAD 1000^TM^ Thermal Cycler (Bio-Rad Laboratories, Hercules, CA, USA) in a total volume of 50 μL of PCR mixtures containing TaKaRa Taq^TM^ DNA polymerase (Takara Bio, Qingdao, China, Code No. R001B) at 1.25 U, 10× PCR buffer (Mg^2+^ Plus) at 5 μL, dNTP mixture (0.01 μM each), 1 μM of each primer, 100 to 500 ng of DNA template and ultrapure water added up to 50 μL. The PCR products were verified by staining with Ethidium Bromide after separation on 2% agarose electrophoresis gels and purified using an E.Z.N.A Gel Extraction kit (Omega Bio-tek, Norcross, GA, USA). Amplicons were sequenced by MEGA Company, Guangzhou, China.

SeqMan V.5.00 (DNASTAR, Madison, WI, USA) was used to assemble sequences generated from forward and reverse primers. The confirmed *ITS*, *tub2*, *tef1-α*, *LSU* and *SSU* DNA sequences of the five isolates were deposited in NCBI with GenBank accession numbers listed in Table S2. Sequence similarities were determined using Blastn program (http://www.ncbi.nlm.nih.gov/BLAST/Blast.cgi), and sequences from known species in the same genus were downloaded from the NCBI database (Accession nos. are listed in Table S3). Joint sequences of two gene regions (*ITS* plus *tub2* for E1, F7, L3 and P4, and *ITS* plus *LSU* for N9) were aligned with ClustalW. Ambiguously aligned regions were excluded and gaps were treated as missing data. Estimation of evolutionary divergence between strain sequences was conducted using the Tamura–Nei model, and phylogenetic trees were constructed using MEGA 6.0 by the Maximum-Likelihood method with 1000 bootstrap replications. Sequences from type, representative or paper-published strains are also included as references listed in Table S3.

### 2.5. Pathogenicity Tests on Detached Leaves and Branches

Healthy bayberry leaves (cv. Dongkui) and branches (cv. Qingdi) (15–20 cm) were detached from trees and surface-sterilized with 5% NaClO for 1 min and washed with sterile water three times. A cross was made on the back of a leaf or branch with a sterile inoculation needle. A mycelial plug (5 mm in diameter) was punched out from the edge of a 3-day-old colony and placed onto the center of the cross with the mycelia facing downwards. Treated leaves were then placed on wet sterilized filter paper in petri dishes (13 × 13 cm), while the treated branches were put in sterilized glass bottles containing sterilized water, with inoculation sites covered with wet sterile degreasing cotton. All the treatments were kept in a growth chamber with controlled conditions of 28 ± 2 °C, 75% ± 15% relative humidity and 12 h white light illumination (illuminance 7350 lx, 12,250 cd, LED light, Artificial Climate Chamber MGC-450BP-2, 2000 W input power, Shanghai YiHeng Scientific Instruments Co., Ltd). Blank PDA plugs without mycelia were used as controls. Leaves and branches were visually examined after 9 days and the sizes of symptomatic areas on the leaves and the lengths of brownish on branches were recorded and measured using Image J. The experiment was performed in triplicates three times. All column figures were produced using GraphPad Prism 7.0 (GraphPad Software, San Diego, CA, USA).

To fulfill Koch’s postulates, the fungi were re-isolated from the infected leaves and twigs, and identified on the basis of colony and conidial morphology accompanied with *ITS* sequencing.

### 2.6. Indoor Fungicide Screening

Fungicides with low toxicity (acute oral LD50 for rat >500 mg/kg, acute dermal LD50 for rat >2000 mg/kg and inhalation LC50 (4 h) for rat >2.0 mg/L) used in this study are: Pyraclostrobin, Mancozeb, Prochloraz (copper salt), 15% Difenoconazole + 15% Propiconazole, Iprodione, Thiophanate-Methyl, Tebuconazole, Difenoconazole, Azoxystrobin, Chlorothalonil, Myclobutanil, Matrine, Boscalid, Hymexazol, Dithianon and Carbendazim (for product names, manufacturer and recommended concentration for field application, see Table S4). They were diluted in series with the middle concentration as 10^−3^ folds of the recommended concentration for field application listed in Table S2, and added into PDA medium for growth inhibition assay. Mycelial plugs (5 mm in diameter) were punched out from the edge of a 5-day-old fungal colony and placed onto the center of the toxic plates. Controls were set with blank PDA plugs without mycelia. All plates were incubated in darkness at 28 °C until mycelia developed to the edge of the control plates. Pictures were taken and diameters of all the fungal colonies were measured. The growth inhibition rate was calculated from mean values as: Inhibitory rate (%) = (mycelial diameter in control − mycelial diameter in toxic plate)/mycelial diameter in control × 100% [20]. The half maximal effective concentration (EC_50_) was calculated using GraphPad prism 7.0.

### 2.7. Tests of Antagonistic Bacteria against Pathogenic Fungi

To screen the antagonistic bacteria for biocontrolling the disease, we collected plant rhizospheric soils from Guangdong, Guangxi and Sichuan Provinces. Each of the 4 g fresh soil was added into 20 mL of sterile water and cultured at 180 rpm, 28 °C for 20 min. One hundred microliters were spread onto LB (per liter contains 10 g of tryptone, 5 g of yeast extract and 10 g of NaCl) agar (1.5% *w*/*v*) plates and incubated in darkness at 28 °C for 24 h. Single colonies were picked for testing their inhibitory activities against the pathogenic fungi using the plate confrontation method. Briefly, mycelial plugs (5 mm in diameter) were punched out from the edge of a 5-day-old fungal colony and placed onto the center of PDA plates. Biocontrol bacteria and *Escherichia coli* DH5α were respectively cultured in LB medium overnight, 1 μL of which was spotted onto the plate 1.5 cm away from the plate edge, with two spots for one bacterium. All the plates were incubated at 28 °C until mycelia developed to cover the colonies of *E. coli* DH5α. The antibiosis activities were determined by comparing the inhibition distances between the antagonistic bacteria to the fungal colony and the *Es. coli* DH5α to the fungal colony.

We also tested the antibiosis activity of zeamines produced by EC1 against the five pathogenic fungi isolated from bayberry. Briefly, EC1 strain was cultured in LS5 medium [18], and 1, 5 and 10 mL of its supernatants (OD_600_ = 1.8) were respectively added into 100 mL of PDA medium for growth inhibition assay. Mycelial plugs (5 mm in diameter) were punched out from the edge of the 5-day-old fungal culture and placed onto the center of the toxic plates. Controls were set as 10% of ΔzmsA (*zmsA* gene deletion EC1 mutant which could not produce zeamines [13]) supernatant was added into the PDA medium. All plates were incubated at 28 °C until CK mycelia developed to the edge.

### 2.8. Statistical Analysis

Statistical analysis on control efficacy using fungicides or biocontrol agents was performed by a one-way analysis of variance (ANOVA). The EC_50_ of fungicides was calculated by ED50plus v1.0 (written by Mario H. Vargas, Instituto Nacional de Enfermedades Respiratorias, Mexico City, Mexico). Multiple comparisons were performed by the Least Significant Difference (LSD) method (α = 0.05). Same letters denote non-significant and different letters denote significant differences.

## 3. Results

### 3.1. Incidence and Symptoms of Bayberry Twig Blight in Jinzao Town

Since different cultivars were mix-planted in fields, almost all the plantations were susceptible to the disease with varying degrees in different cultivars. The total incidence rate in fields was about 84%; amongst them, the incidence was 91% for Dongkui, the highest, and about 80% for Fujian Dahong and Yingsi, whereas significantly it was lower for the local varieties such as Wusu, Aodi Wusu, Qingdi and Shanmei, ranging from 15% to 33% (Table 1). Generally, the disease was more serious in old plantations and comparably lighter in newly developed planting orchards. Exotic varieties were more susceptible than local varieties, but the incidence rate of the latter has also been gradually increasing.

**Figure 1:**
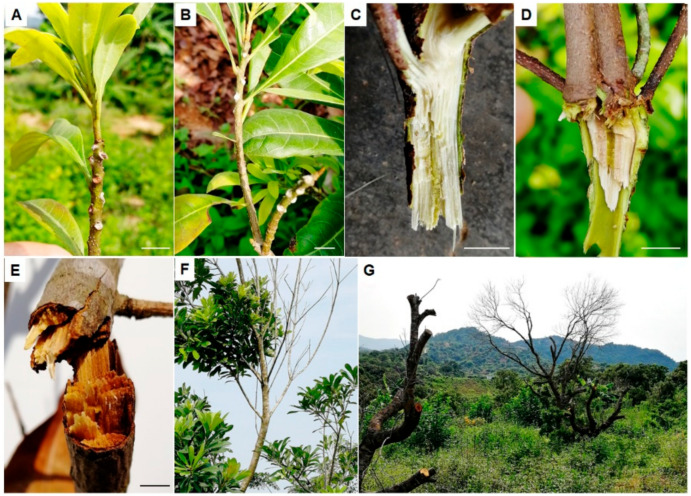
Symptoms of bayberry twig blight disease in Jinzao town. (**A**) and (**B**), white mycelia on petiole bases in young shoots; (**C**), healthy twig with light green interior; (**D**), newly diseased twigs with interior turning brown; (**E**), late-onset twig completely turning brown and dry; (**F**), defoliation of the infected trees; (**G**), bald shoots and trunks. Bar = 1 cm.

Upon disease attack, young shoots firstly became yellow, brown, and then, scorched with leaves drooping down, followed by the appearance of mildew spots on leaf scars, petiole bases or wounded twigs after fruit harvest or typhoon transits (Figure 1A,B). Compared with the healthy twigs (light green) (Figure 1C), the interior of the infected twigs turned brown (Figure 1D). Subsequently, symptoms spread down rapidly to the branches and trunk, obstructing water delivery and resulting in twigs and branches becoming dry and withered (Figure 1E) when the temperature was over 25 ℃. All leaves, whether new or old, defoliated from the branches and bald shoots were common in the fields (Figure 1F,G). Finally, many crowns of severely ill trees had to be cut down (Figure 1G), resulting in serious yield losses.

### 3.2. Pathogen Isolation and Identification

Due to the similar symptoms of diseased trees in different plantations, we randomly selected 7 trees from two separate bayberry orchards (one was 4 years old and the other was 12 years old) in Jinzao town in a distance of 800 m for pathogen isolation. As a result, a total of 52 fungal isolates were obtained from the diseased twigs and leaves of bayberry cvs. Yingsi, Dongkui and Fujian Dahong collected from the two orchards. All the isolates were identified based on their housekeeping gene sequences of *ITS*, *tef1-α* and *tub2*, and divided into 7 different taxonomic groups according to their sequences of the same gene, which were *Colletotrichum gloeosporioides*, *Epicoccum sorghinum*, *Neofusicoccum parvum*, *Lasiodiplodia theobromae*, *Nigrospora oryzae*, *Pestalotiopsis* sp. and *Cytospora tibouchinae*, respectively (Table 2). Among them, the majority (14) of the isolates were *Pestalotiopsis* sp., ten were *C. gloeosporioides*, nine were *Ni. oryzae*, and the remaining belonged to *E. sorghinum*, *L. theobromae*, *N. parvum* and *Cy. tibouchinae* (Table 2). *E. sorghinum* was only isolated from the petiole base of cv. Yingsi in Caisong Peng Orchard, *L. theobromae* was found on leaves from cv. Fujian Dahong, petiole bases and interior branches from cv. Yingsi in Caisong Peng Orchard, and *Cy. tibouchinae* was only isolated from the Limin Orchard (Table 2). 

### 3.3. Fungal Isolates in E, F, L, N and P Groups Are Pathogenic

**Figure 2:**
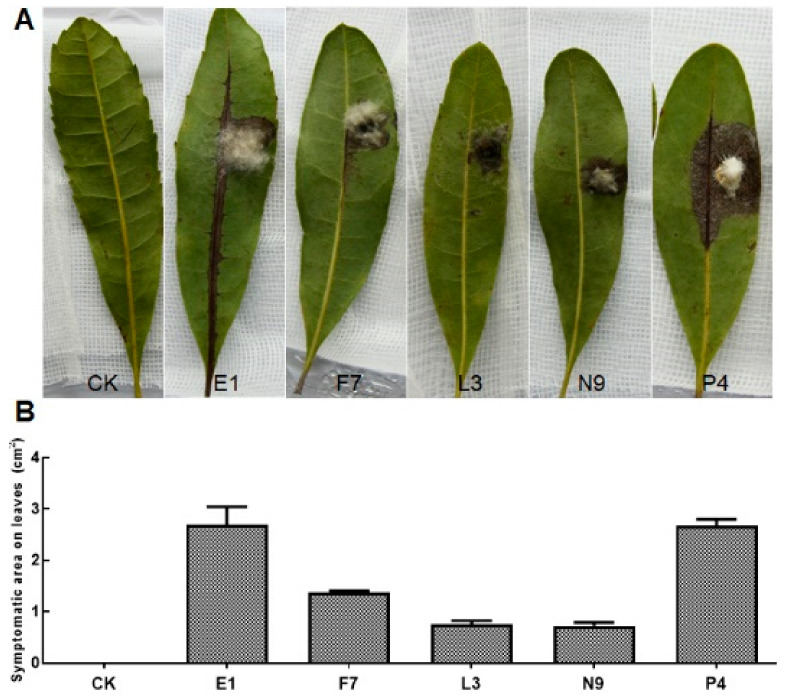
Pathogenicity tests of fungal isolates on detached bayberry leaves. (**A**) Symptoms on detached leaves causing by the inoculated fungal isolates; (**B**) the sizes (cm^2^) of the symptomatic area measured by Image J. Isolates were inoculated on healthy leaves collected from healthy plants (cv. Dongkui) after surface-sterilization. Treated leaves were then placed on wet sterilized filter paper in petri dishes (13 × 13 cm), and kept in a growth chamber with controlled conditions of 28 ± 2 °C; 75% ± 15% relative humidity and 12 h white light (12,250 cd) illumination for a week. Blank PDA plugs without mycelia were used as controls (CK, control check). Leaves were visually examined after 9 days and sizes of symptomatic areas on the leaves were recorded and measured using Image J. The experiment was repeated in triplicates three times. Fungal isolates causing no disease symptoms were not displayed.

**Figure 3:**
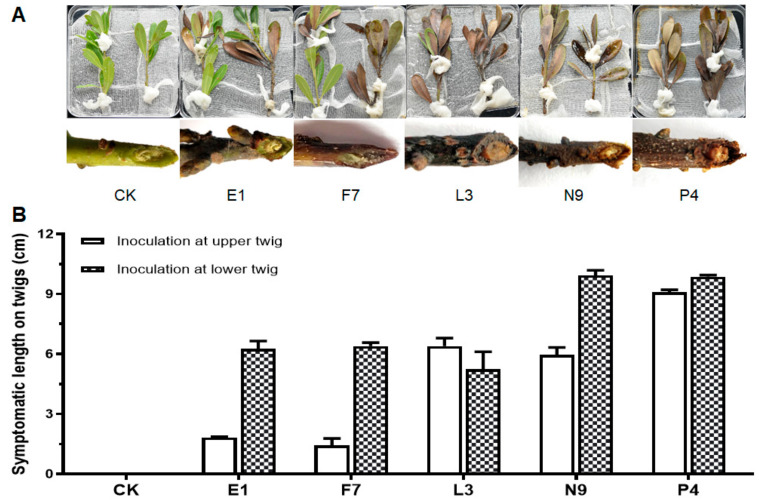
Pathogenicity tests of fungal isolates on detached bayberry branches. (**A**) The lesions on leaves and interior of branches caused by inoculated fungal isolates; (**B**) the symptomatic lengths on inoculated upper and lower twigs. Healthy bayberry branches collected from healthy plants (cv. Qingdi) (15–20 cm) were detached from trees and surface-sterilized. A cross was made on a branch (at the upper site or the bottom site) and a mycelial plug (5 mm in diameter) was placed onto the center of the cross with mycelia facing down. The inoculation sites of the treated branches were covered with sterile wet degreasing cotton, put in sterilized glass bottles with sterilized water, and then kept in a growth chamber with controlled conditions of 28 ± 2°C; 75% ± 15% relative humidity and 12 h white light (12,250 cd) illumination for a week. Blank PDA plugs without mycelia were used as controls (CK). The experiment was repeated in triplicates three times. Fungal isolates caused no disease symptoms were not displayed. Photos were taken after 9 days and the symptomatic length on twigs was measured.

After pathogen isolation and purification, we first performed pathogenicity tests on detached healthy leaves and branches. According to the ITS, *tef*1-α and *tub2* sequences of all the 52 isolates, they were divided into 7 species. For pathogenicity tests, one isolate of the same species from each tree was selected (Table 2). The results showed that among the inoculated isolates, all the E, F, L, N and P isolates caused variously sized disease symptoms, whereas isolates from C, U and V groups could not result in disease. E1, F7, L3, N9 and P4 were then chosen as the representative isolates for further identification and pathogenicity tests. Specifically, leaves inoculated with isolate P4 turned brown first at the inoculation sites on the 3rd day, those inoculated with isolates E1 and F7 became brown on the 4th day and lesions caused by E1 expanded and developed downward along the veins; leaves inoculated with isolates L3 and N9 showed discoloration after about one week; on the 9th day, lesions on leaves caused by E1 and P4 were comparably larger than those by the others (Figure 2A,B). For detached shoots, discoloration spread upward along the twigs and leaves from the inoculated bases, with symptoms appearing in the order as P4 on the 3rd day, E1, F7, L3 and N9 on the 4th day (Figure 3A,B). On the 7th day, those detached branches inoculated with isolate P4 lost most of their leaves. Finally, all branches turned brown and scorched, and leaves shed on the 9th day. Both xylem and phloem showed browning in oblique sections, while those in CK kept healthy and green with no diseased symptoms observed (Figure 3A). Pathogens were then re-isolated from the diseased tissues and cultured on PDA plates, and then, identified by microscope observation and *ITS* sequencing. The results showed that they had the same characteristics of colonial and microscopic morphologies as well as *ITS* sequences as the ones used for inoculation. In summary, we can conclude that fungal isolates of E1, F7, L3, N9 and P4 are the pathogens causing bayberry twig blight disease in Jinzao town.

### 3.4. Characterization of Pathogens E1, F7, L3, N9 and P4

To identify the taxonomic status of the pathogenic isolates E1, F7, L3, N9 and P4, we cultured them on four different types of media, including PDA, MEA, OA and WA for the sake of generating fruiting bodies for microscopic observation, and extracted their genomic DNAs for MLSA on *ITS*, *tub2*, *tef1*-α, *LSU* and *SSU* genes.

#### 3.4.1. Characterization of E1

**Figure 4:**
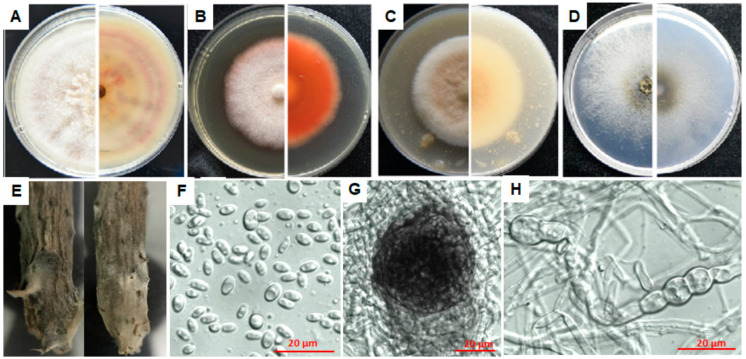
Morphological characteristics of *Epicoccum sorghinum* isolate E1. (**A**–**D**) The front and back of the colony morphology on PDA, MEA, OA and WA plates; (**E**), hyphae on infected twigs; (**F**), conidia were taken from sterile bayberry twigs on WA plates after 7 days of culture; (**G**), pycnidium; (**H**), chlamydospore. Photos were taken by a Nikon D750 after 7 days of culture. Fungal mycelia and spores were observed and photographed with a Zeiss Axio observer Z1 Microscope (Carl Zeiss Microscopy, Germany).

According to morphological and molecular characterization, isolate E1 was identified as *Epicoccum sorghinum* (Basionym: *Phyllosticta sorghina* = *Phoma sorghina* = *Epicoccum sorghi* = *Phoma insidiosa*). On PDA, MEA and OA plates, *E. sorghinum* E1 grew slowly and took more than 7 days to grow to the edge of the petri dish; upper mycelia were cottony or velvety; light purple concentric rings appeared on the reverse side of the colony on the PDA plate, with orange blocks near the colony center (Figure 4A). The color of the colony on the MEA plate was less variable with the upper layer white to light salmon at the front side, and salmon on the reverse side (Figure 4B). On the OA medium, the mycelia in the center of the colony were light pink, flocculent, while the rest were white, cushioned, and the center on the back was purple; the colony on the WA plate was white and powder-like (Figure 4C). Conidia were taken from sterile and dry bayberry branches on the WA plates after inoculation for 7 days (Figure 4D,E), which were colorless, non-septate, elliptic, 4.9–7.7 μm ($\bar{x}\;$= 6.00 ± 0.84 μm) of length and 2.6–5.8 μm ($\bar{x}\;$= 3.55 ± 0.67 μm) of width (Figure 4F) with L/W ratio = 1.7 (*n* = 30). Chlamydospores were easy to form, 5.8–13.6 μm ($\bar{x}$ = 8.89 ± 2.40 μm) × 4.1 – 7.5 μm ($\bar{x}\;$= 5.08 ± 1.23 μm) (*n* = 20) (Figure 4H), and the pycnidia were round, 49.60–55.22 μm ($\bar{x}\;$= 52.82 ± 2.16 μm) (*n* = 20) in diameter (Figure 4G).

Compared with *Epicoccum* species in the GenBank database, isolate E1 was mostly similar to *E. sorghinum*, with 99.82% identity (100% coverage value) to isolates P8, P6, 325p and GZDS2018BXT010^T^ in *ITS* sequence, 98.84% identity to isolates BJ-F1 (100% coverage value) and H10 (96% coverage value), as well as 98.04% identity (88% coverage value) to GZDS2018BXT010^T^ in *tub2* sequence, and 99.93% identity (99% coverage value) to GZDS2018BXT010^T^ in *LSU* sequences. Moreover, it was respectively 98.63% and 99.91% similar to *Phoma* sp. isolate FJ-1 in *tef1-α* sequence (87% coverage value) and isolates FN40 and FJ-2 in *SSU* sequence (100% coverage value). Phylogenetic analysis based on the joint *ITS*+*tub2* sequences was performed since there are few sequences of the other three genes in *Epicoccum* genus released in the database. The results showed that isolate E1 was in a clade containing *E. sorghinum*, *E. viticis*, *E. camelliae* and *E. latusicollum* (Figure S1). Although these strains were clustered in the same clade, *E. sorghinum* can be easily distinguished from the other three species by its colonial morphology. The colony center of *E. viticis*, *E. camelliae* and *E. latusicollum* on OA, MEA and PDA is gray to grayish dull green on the front [21], buff or hazel on the back, while of *E. sorghinum* it is respectively light pink on OA (purple on the back), salmon or reddish-brown on MEA and cottony with light purple concentric rings on PDA on the back [21,22].

In view of its highest homology of *ITS*, *tub2* and *LSU* gene sequences to *E. sorghinum* and the colonial morphology, we named E1 as *Epicoccum sorghinum*.

#### 3.4.2. Characterization of F7

**Figure 5:**
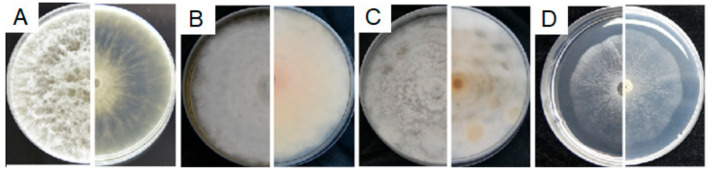
Conidial morphology of *Neofusicoccum parvum* isolate F7. (**A**–**D**) The front and back of the colony morphology on PDA, MEA, OA and WA plate.

Isolate F7 failed to form spores even by induction means of lacerating colonies, ultraviolet exposure and host branch inoculation. It grew fast and covered fully on the dishes in 3–5 days on PDA, MEA and OA media, first producing plenty of white long aerial hyphae on the PDA plates which then turned greenish or black after 7–10 days (Figure 5A). Colonies on the MEA and OA plates were cottony initially with shorter aerial hyphae which turned deep green or black after 7–10 days (Figure 5B,C). It grew comparably slower with white, short and sparse hyphae appressing on the WA plates (Figure 5D). 

The *ITS* sequence of isolate F7 was 100% and 97.28% identical to that of *N. parvum* ATCC 58191^T^ and CMW9081^T^ with 92% and 97% coverage values, respectively. Additionally, its *tub2* and *SSU* sequences were 99.77% and 99.9%, similar to those of isolate CMW9081 with 93% and 100% coverage values, respectively; whereas, it was 97.19% and 99.70%, similar to *Diplodia corticola* BKCO1_4100086 in *tef*1-α sequence and *N. ribis* AFTOL-ID 1232 in the *LSU* sequence with 100% and 97% coverage values, respectively. Phylogenetic analysis of isolate F7 based on the joint *ITS*+*tub2* sequences showed that it was grouped in the same branch of *N. parvum*, far from the *N. ribis* strain (Figure S2), suggesting that isolate F7 is *Neofusicoccum parvum* (Basionym: *Fusicoccum parvum = Botryosphaeria parva*).

#### 3.4.3. Characterization of L3

**Figure 6:**
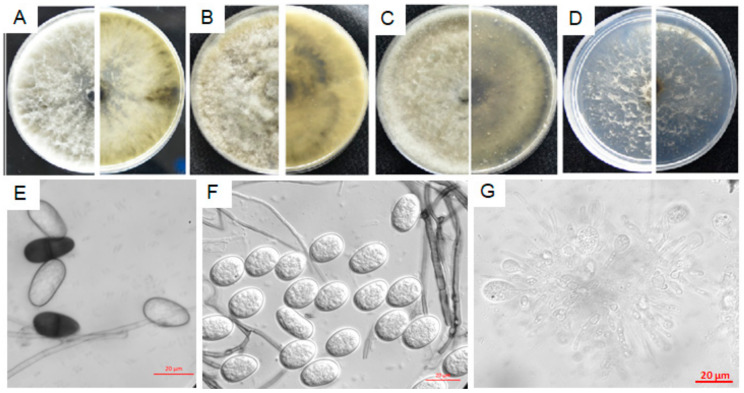
Morphological characteristics of *Lasiodiplodia theobromae* isolate L3. (**A**–**D**) The front and back of the colony morphology on PDA, MEA, OA and WA plate; (**E**), young (transparent) and mature (dark) conidia with one septum, induced by ultraviolet radiation for 7 days on bayberry twigs on WA plates; (**F**), transparent young conidia; (**G**), pycnidium. Fungal mycelia and spores were observed and photographed with a Zeiss Axio observer Z1 Microscope (Carl Zeiss Microscopy, Germany).

Colonies of isolate L3 on PDA, MEA and OA plates (Figure 6A–C) were similar to those of isolate F7 (Figure 5), while white and flocculent on the WA plates (Figure 6D), growing rapidly on the PDA plates, reaching 9 cm in diameter within 5–7 days. Combining morphological and molecular characterization, isolate L3 was identified as *Lasiodiplodia theobromae* (Basionym: *Lasiodiplodia theobromae* = *Diplodia theobromae*, *Botryodiplodia theobromae*), belonging to *Botryosphaeriaceae* as isolate F7. Conidia were collected from bayberry twigs on the WA plates after ultraviolet exposure for 7 days. Developing young conidia (Figure 6E–G) were oval, hyaline and non-septa, while old conidia became dark brown to black with one septum (Figure 6E), 22.86–28.02 μm ($\bar{x\;}$= 25.42 ± 1.46 μm) in length and 10.43–15.91 μm ($\bar{x}\;$= 12.89 ± 1.13 μm) in width with L/W ratio = 2 (*n* = 30). Conidiogenous cells were hyaline, cylindrical to sub-obpyriform (Figure 6G).

Compared with all *Lasiodiplodia* species in the GenBank database, isolate L3 was the same as *L. theobromae* strains EucN188 and CBS 111530, and 99.63% identical to *L. theobromae* CBS 164.96^T^ in *ITS* sequence, with 100%, 100% and 94% coverage values, respectively; 100%, 100% and 99.72% identical to *L. theobromae* CBS 164.96^T^ in *tub2*, *LSU* and *SSU* sequences with 83%, 43% and 100% coverage values, respectively; 96.49% identical (100% coverage value) to *D. corticola* BKCO1_4100086 in *tef1-α* sequence. Phylogenetic analysis of isolate L3 based on the joint *ITS*+*tub2* sequences indicated that it was grouped in the same branch of *L*. *theobromae* and *L. egyptiacae*, closer to the former (Figure S3). *L. egyptiacae* is morphologically and phylogenetically closely related to *L. theobromae*, but can be distinguished based on its smaller conidia (17–27 × 11–13 μm) [23]. In combination of the morphological and genetic characteristics, we named isolate L3 as *Lasiodiplodia theobromae*.

#### 3.4.4. Characterization of N9

**Figure 7:**
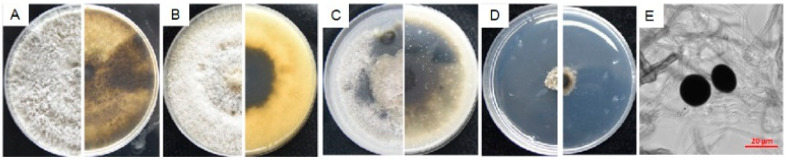
Morphological characteristics of *Nigrospora oryzae* isolate N9. (**A**–**D**) The front and back of the colony morphology on PDA, MEA, OA and WA plate; (**E**), conidia induced by ultraviolet radiation for 7 days on bayberry twigs on WA plates. Fungal mycelia and spores were observed and photographed with a Zeiss Axio observer Z1 Microscope (Carl Zeiss Microscopy, Germany).

Colonies of isolate N9 were colorless at first, then dark brown, velvety with no aerial hyphae on PDA, MEA and OA media (Figure 7A–C), growing rapidly, reaching 9 cm in diameter within 5 d, woolly at the front and pale yellow on the reverse in 3–5 days, white to grey to black at the front and dark brown to black on the reverse in 5–7 days. Compared with other isolates, hyphae of N9 were unable to grow on the WA plates (Figure 7D). Conidia collected from bayberry twigs on the WA plates after ultraviolet exposure for 7 days were black, non-transparent, spherical, 10.98–22.62 μm ($\bar{x}$ = 18.38 ± 2.27 μm) in length and 13.02–21.40 μm ($\bar{x}$ = 18.22 ± 2.04 μm) in width with L/W ratio = 1 (*n* = 30) (Figure 7E,F).

Isolate N9 was the same as *Nigrospora* sp. strain KH00291 in *ITS* sequence, homologous to *Ni. oryzae* with 99.85% identity (98% coverage value) to isolate AFTOL-ID 2179 in *LSU* sequence, respectively 99.81% and 99.71% identity to isolates IFO 32860 and CBS 480.73 in *SSU* sequence both with 98% coverage value, shared low homology to *N. osmanthi* (92.26% and 72.31% identical to HN1701 in *tub2* sequence and *tef1-α* sequences with 100% and 82% coverage values, respectively) due to the lack of sequences released. Thereafter, phylogenetic analysis of isolate N9 was performed based on the joint *ITS*+*LSU* sequences, indicating that it was grouped in the same branch of *Nigrospora* sp. KH00291, *Ni. Osmanthi*, *Ni. Guilinensis*, *Ni. lacticolonia* and *Ni. oryzae* (Figure S4). Although these strains were clustered in the same clade, N9 can be distinguished from the other three species by its colonial morphology. Compared with N9, colonies of *Ni. guilinensis* and *Ni. osmanthi* grow much slower on the PDA plates, needing 14 days and 10 days respectively to reach 9 cm in diameter [24]. Colonies of *Ni. lacticolonia* reach 9 cm diameter in 6 days on the PDA plates, floccose, surface and reverse creamy white, with dark brown patches on the reverse side; conidia slightly ellipsoidal, spherical 11.5–16.5 μm diam, ellipsoidal 13.5−17.5 × 10.5−13.5 μm [24]. 

Given that it shared high similarity with *Ni. oryzae* on both morphological and molecular characteristics, we classified it as *Nigrospora oryzae.*

#### 3.4.5. Characterization of P4

Isolate P4 was identified as a new species, *Pestalotiopsis myricae*. Description and illustration were indicated below.

Taxonomy of Pestalotiopsis myricae

*Pestalotiopsis myricae* Jianuan Zhou and Wenjun Li, sp. nov.

Mycobank no.: MB 834865

**Figure 8:**
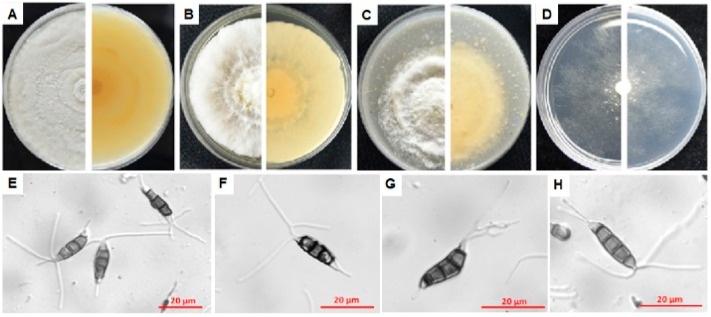
Morphological characteristics of *Pestalotiopsis myricae* isolate P4. (**A**–**D**), the front and back of the colony morphology on PDA, MEA, OA and WA plate; (**E**–**H**), conidia with two (**E**), three (**E**,**F**,**H**) or four (**E**,**G**) tubular apical appendages, and one (**E**–**G**) or two (**H**) appendages at the basal cell. Conidia were generated on PDA plates after 7 days of culture. Fungal mycelia and spores were observed and photographed with a Zeiss Axio observer Z1 Microscope (Carl Zeiss Microscopy, Germany).

Conidia were straight or slightly curved, clavate-fusiform and 17.6–26.4 μm ($\bar{x}$ = 21.07 ± 2.10 μm) × 3.7–6.9 μm ($\bar{x}$ = 5.40 ± 0.68 μm) with L/W ratio = 3.9 (*n* = 35); basal cell long and conic, hyaline, thin, verruculose and 3.1–5.0 μm ($\bar{x}$ = 3.80 ± 0.48 μm) (*n* = 30) long, containing 1–2 (mostly 1) basal appendages with length between 4.9–12.5 μm ($\bar{x}$ = 7.06 ± 1.46 μm) (*n* = 30) (Figure 8E–H); three middle cells doliform, dark brown to olivaceous, almost concolorous and verruculose, 3.2–5.8 μm ($\bar{x}$ = 4.42 ± 0.66 μm) (*n* = 30) long for each; apical cell hyaline, conical, 3.0–5.5 μm ($\bar{x}$ = 4.32 ± 0.66 μm) (*n* = 30) long with 2–4 (mostly 2) tubular apical appendages arising from the apical crest, flexuous, ranging from 18–40.2 μm ($\bar{x\;}$= 26.70 ± 5.52 μm) (*n* = 30).

Culture characteristics: Colonies on PDA, MEA and OA media had white aerial mycelium on surface with undulated edge and pale luteous on the reverse side (Figure 8A–C). Colonies on the MEA plates were more cottony than those on the PDA plates, and had shorter aerial hyphae on the OA plates, being translucent and thin on the WA plates (Figure 8A,C,D). Fruiting bodies were black pellets and easily observed by eyes after growing on the PDA plates for 7–10 days.

Habitat: on *Myrica rubra*.

Holotypus: *Pestalotiopsis myricae* P4, deposited in Integrative Microbiology Research Center, South China Agricultural University, Guangzhou, China.

Known distribution: China.

Isolate P4 was the same (100% coverage value) as *Pestalotiopsis* sp. strains LB11, LB12 and LB13, *P. maculans* strain M17, *P. sydowiana* strain xsd08016 and *P. microspora* strain XSD-42 in *ITS* sequence, 98.74% identical (100% coverage value) to *P. paeoniicola* in *tub2* sequence, 92.55% (94% coverage value) to *Pestalotiopsis* sp. HGUP4077 in *tef1-α* sequence, 98.80% (100% coverage value) to *P. microspora* strain CWK1114 in *LSU* sequence, 99.81% and 99.72% (100% coverage value) to *Pestalotiopsis* sp. isolate YN-6 and *P. microspora* isolate WT98 respectively in *SSU* sequence. Phylogenetic analysis of isolate P4 based on the joint *ITS* and *tub2* sequences showed that it was clustered with many *Pestalotiopsis* species such as *P. conigena, P. albomaculans, P. crassiuscula, P. gracilis* and *P. versicolor* (Figure S5), unable to be classified in a specific species.

To distinguish isolate P4 from the others in the same phylogenetic branch, morphological characteristics of the conidia were compared in Table 3. Specifically, the median cells of *P. conigena*, *P. albomaculans, P. gracilis* and *P. versicolor* were versicolorous [25,26,27,28,29,30], while those of isolate P4 and *P. crassiuscula* were concolorous [31]; the length of apical appendages of *P. crassiuscula* (10–23 μm) was greatly shorter than P4 (18–40.2 μm) and the number of apical appendages of *P. crassiuscula* was mostly 3 [31], while which of P4 was mostly 2.

Therefore, in view of the special morphological characteristics (especially the lengths of apical and basal appendages, and the occasional 2 basal appendages) of P4, we classified it as a new species in genus *Pestalotiopsis*, naming it as *Pestalotiopsis myricae.*

### 3.5. Prochloraz (Copper Salt) Is One of the Most Promising Fungicides That Could Control the Growth of the Five Pathogens

In order to give some technical guidance for field application, we screened low toxic fungicides and biological control agents with high efficiency. The results of the indoor toxicity test showed that among the 16 tested fungicides, Prochloraz (copper salt) had good controlling efficiency against all of the five pathogens, especially against F7 (EC_50_ = 0.0037 mg/L, 100% inhibition under 4.25 mg/L), N9 (EC_50_ = 0.0004 mg/L, 100% inhibition under 4.2 mg/L) and P4 (EC_50_ = 0.0692 mg/L, 92.15% inhibition under 4.25 mg/L) (Figure S6, Table 4 and Table 5). In addition, Pyraclostrobin also had good efficiency on controlling four of the five pathogens, including E1, F7, N9 and P4, especially F7 (EC_50_ = 0.0161 mg/L, 92.68% inhibition under 2.5 mg/L) and P4 (EC_50_ = 0.0088 mg/L, 100% inhibition under 2.5 mg/L) (Figure S6, Table 4 and Table S5). The 15% Difenoconazole + 15% Propiconazole and Difenoconazole had controlling efficiency on three of the five pathogens (Figure S6, Table 4 and Table S5). Surprisingly, Mancozeb, Thiophanate-Methyl, Hymexazol and Carbendazim, the most commonly used fungicides in fields, had no good fungistatic effect on the five pathogens, and another common fungicide Chlorothalonil had only moderate controlling effect against F7 and P4 (Figure S6, Table 4 and Table 5).

### 3.6. Bacillus Velezensis Strain 3–10 and Zeamines Can Be Used as Potential Antifungal Agents to Biocontrol the Bayberry Twig Blight

**Figure 9:**
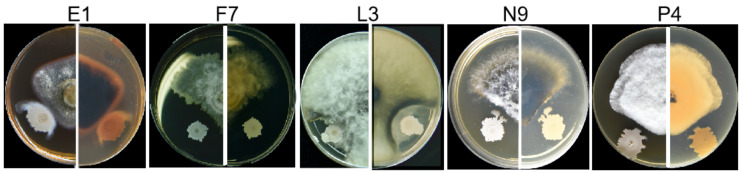
Inhibition effects of *Bacillus velezensis* 3–10 on pathogens E1, F7, L3, N9 and P4. Mycelial plugs (5 mm in diameter) were punched out from the edge of the 5-day-old fungal culture and placed onto the center of PDA plates. Biocontrol bacteria were cultured in LB and were adjusted to OD_600_ = 1.5, 1 μL of which was spotted onto the plate 1.5 cm from the plate edge. All plates were incubated at 28 °C until mycelia developed to the edge of the plates.

In order to explore a diverse and safer strategy for controlling the bayberry twig blight disease, we screened antagonistic bacteria from soils and used zeamines produced by the rice foot rot pathogen *D. zeae* EC1 to test their inhibitory activities against the five pathogens. The results showed that a patent biocontrol bacterium named as *Bacillus velezensis* strain 3–10 (identified in our lab based on the *16S rDNA*, *gyrA* and *gyrB* sequences with accession nos. MN515140, MN648416 and MN648417, respectively) could inhibit all of the five pathogenic isolates with obvious growth inhibition zones around it (Figure 9), and zeamines achieved markably amazing inhibition effect (94.02% to 100%) on all of the five isolates, even when the EC1 supernatant took up 10% of the medium (Table 5 and Figure S7).

## 4. Discussion

In this study, we identified five species of fungi including *Epicoccum sorghinum*, *Neofusicoccum parvum*, *Lasiodiplodia theobromae*, *Nigrospora oryzae* and *Pestalotiopsis myricae* (new species) as the pathogens responsible for bayberry twig blight disease in Jinzao town, Shantou City. Among them, *Epicoccum sorghinum* was only isolated from the petiole base where white mycelia grew in abscission zone on young shoots, while the other four pathogens were obtained from both symptomatic leaves and the interior of diseased branches (Table 2). From the speed and degree of pathogenesis on both detached bayberry leaves and branches, *P. myricae* and *E. sorghinum* showed stronger aggressiveness than the other three pathogens (Figure 2 and Figure 3).

*E. sorghinum* is a kind of cosmopolitan fungus and facultative plant pathogen with preference for Poaceae, especially in tropical regions [32]. It is one of the major components of sorghum grain-mold disease complex [22] and has been reported as a cause of leaf spot disease in different types of plants, such as maize, wheat, tobacco and *Bletilla striata* [33,34,35,36]. *N. parvum* and *L. theobromae,* both belonging to Botryosphaeriaceae, are either pathogens, endophytes or saprobes mainly on woody hosts [23]. Blueberry (*Vaccinium corymbosum)* stem blight, caused by *Botryosphaeria dothidea*, *N. parvum* and *L. theobromae*, is the most destructive disease affecting blueberry production and quality worldwide [11]. Typical symptoms of blueberry stem blight are similar with those of bayberry, including twig wilting, leaf chlorosis and necrosis on individual branches with hyphal expanding into plant crowns, resulting in systemic infection of vascular tissue and eventually mortality [37,38]. *Nigrospora* is an important genus of fungal ascomycetes with a cosmopolitan distribution and a wide host range, which has also been commonly recorded as pathogens on many important economic crops, fruits and ornamentals [24], e.g., stem blight on *Brassica juncea* caused by *Ni*. *oryzae* in India [39] and leaf blight on *Camellia sinensis* caused by *Ni*. *sphaerica* in China [40]. To our knowledge, this is the first study reporting *E. sorghinum*, *N. parvum*, *L. theobromae* and *Ni*. *oryzae* as the pathogens responsible for bayberry twig blight.

In nature, most species of *Pestalotiopsis* are endophytes and saprophytes, some have been shown to be pathogenic to economically important plants, causing a variety of diseases including canker lesions, shoot dieback, leaf spots, needle blight, tip blight, grey blight, scabby canker, severe chlorosis, fruit rots and various post-harvest diseases [41,42,43,44,45,46], e.g., the macadamia leaf blight caused by *P*. *microspore* in Yunnan Province [47], the blueberry leaf spot and root rot caused by *P. clavispora* in Shandong Province [48]. Compared with those published *Pestalotiopsis* pathogens such as *P. mangiferae*, *P. vismiae*, *P. versicolor* and *P. microspore* isolated from diseased bayberry twigs in Zhejiang Province [2,3,7], *P. myricae* P4 has its unique characteristics including its significantly longer apical appendages (18–40.2 μm) than the others (14–23.6 μm of *P. mangiferae*, 9.4–17.7 μm of *P. vismiae*, 12.9–28.2 μm of *P. versicolor* and 9.7–28.6 μm of *P. microspore*) and relatively longer (4.9–12.5 μm) basal appendages (sometimes containing 2) than the others (2.8–9.08 μm, only 1) (Figure 8). Such characteristics are very different from the known *Pestalotiopsis* species. The *ITS* and *tub2* sequences of isolate P4 are quite different from the strains *P. versicolor* XJ27, XJ42 and RA2-1, and *P. microspora* YS26, YS44 and RA1-2, which caused twig blight disease of bayberry in Zhejiang Province [2]. Isolate P4 shared a higher similarity to *P. versicolor* isolates than to *P. microspora* isolates. For the *ITS* sequence, it was 99.65%, 99.48% and 99.65% respectively identical to isolates XJ27, XJ42 and RA2-1, and 92.39%, 92.39% and 92.19% respectively identical to YS26, YS44 and RA1-2. As for the *tub2* sequence, it shared up to 98.53% identity to the three *P. versicolor* isolates, whereas only 89.80%, 84.05% and 84.05% identity to *P. microspora* YS26, YS44 and RA1-2, respectively. Phylogenetic analysis of the combined *ITS* and *tub2* sequences illustrated that P4 was grouped in the same cluster of *P. conigena, P. albomaculans, P. crassiuscula, P. gracilis* and *P. versicolor* (Figure S5). However, *P. conigena*, *P. albomaculans, P. gracilis* and *P. versicolor* have been no longer classified as *Pestalotiopsis* since 2014 because of their versicolorous median cells [24,25,26,27,28,29,44]. On basis of the unique conidial features, especially the occasionally 2 basal appendages and the longer apical appendages, we regarded P4 as a new species in genus *Pestalotiopsis* and named it as *Pestalotiopsis myricae*.

During disease investigation, we found that chemical control of bayberry twig blight disease was very complicated in fields. Fruit farmers mix-applied varying chemicals including fungicides, even bactericides unreasonably at the same time, even during the fruit harvest period, but failed to obtain good control efficacy. Since the bayberry twig blight in Jinzao town was caused by multiple fungal pathogens including *E. sorghinum*, *N. parvum*, *L. theobromae*, *Ni. oryzae* and *P. myricae,* we tested 16 low toxic fungicides against them. The results indicated that Prochloraz (copper salt) had the best controlling effect on all of the five pathogens, followed by Pyraclostrobin, 15% Difenoconazole + 15% Propiconazole, Difenoconazole and Myclobutanil. 

Reports on the fungicides against *E. sorghinum* are rare, but Rajini et al. tried to find ecofriendly greener alternatives, and in 2019, they found that an extract from *Eclipta alba* had antifungal activity in vitro and in vivo against sorghum fungal pathogen *E. sorghinum* [49]. Our indoor toxicity test showed that the hyphal growth of *E. sorghinum* could be effectively inhibited by Boscalid (EC_50_ = 0.1548 mg/L), followed by Pyraclostrobin, Difenoconazole and Prochloraz (copper salt) (Figure S6, Table 4 and Table S5). 

Several fungicides against *N. parvum* were tested for controlling the blueberry stem blight. The EC_50_ of Mancozeb, Chlorothalonil, Tebuconazole and Thiophanate-Methyl was respectively 4.12, 0.43, 0.45 and 0.35 mg/L in the indoor toxicity test [50]. However, when the pathogen infected the interior of blueberry branch, all fungicide and field medicament controls were ineffective [50]. In our study, no significant inhibition effect of Mancozeb and Thiophanate-Methyl towards *N. parvum* was found in our lab treatment in low concentration (Table 4 and Table 5). Obvious inhibition effect may be observed when the concentration was increased ten times above the recommended concentration for field application.

For *L. theobromae* (maize ear rot), Liu et al. tested 8 fungicides and found that the EC_50_ of Pyraclostrobin, Iprodione, Azoxystrobin, Thiophanate-methyl, Tebuconazole, Difenoconazole and Prochloraz was 0.88, 14.84, 0.23, 5.17, 1.32, 2911.17 and 2.29 mg/L, respectively [51]. However, our results showed that Pyraclostrobin, Azoxystrobin, Thiophanate-methyl and Difenoconazole did not work well in controlling the growth of *L. theobromae* L3 using the recommended concentration for field application (Table 4 and Table S5). After screening the fungicides indoor, we recommend that Prochloraz (copper salt), Iprodione, Tebuconazole, 15% Difenoconazole + 15% Propiconazole and Myclobutanil can be used for field application in controlling the diseases caused by both *N. parvum* and *L. theobromae* (Figure S6, Table 4 and Table S5). 

For *Ni. oryzae*, Azoxystrobin (EC_50_ = 0.0001 mg/L) had the most significant fungal controlling effect, followed by Prochloraz (copper salt), 15% Difenoconazole + 15% Propiconazole, Difenoconazole, Pyraclostrobin and Myclobutanil (Figure S6, Table 4 and Table S5). In a previous study, Azoxystrobin (EC_50_ = 0.009 mg/L) and Chlorothalonil (EC_50_ = 0.4213 mg/L) could inhibit *Ni. oryzae* as well [52].

For the fungicides on controlling *Pestalotiopsis*, Difenoconazole was reported to have the best inhibitory activity on *P. clavispora* (causing blueberry leaf spot and root rot) hyphal growth with 1.97 mg/L of EC_50_, followed by Difenoconazole + Propiconazole, Iprodione, Tebuconazole, Mancozeb and Pyraclostrobin with EC_50_ as 2.70, 5.63, 12.25, 63.22 and 71.10 mg/L, respectively [48]. Whereas our toxicity test results showed that Pyraclostrobin had the best inhibition against the hyphal growth of *P. myricae* P4 with only 0.0088 mg/L of EC_50_ and almost completely inhibited P4 growth under the concentration of 0.25 mg/L, followed by Azoxystrobin, Prochloraz (copper salt) and Chlorothalonil (Figure S6, Table 4 and Table S5). 

On the other hand, the overuse of chemical fungicides for plant disease management has inestimably negative effects, including disease resurgence due to development of resistance in the target pathogens, and environmental and fruit pollutions threatening human and animal health. Therefore, we also explored some promising ecofriendly alternatives of chemical fungicides, and found that a *B. velezensis* strain 3–10 and metabolic zeamines from *D. zeae* EC1—phytotoxins previously identified as potential agents for preventing and controlling fungal pathogens [14,15], could be used as potential biocontrol agents for field application on controlling bayberry twig blight disease (Figure 9 and Figure S7, Table 5).

## 5. Conclusions

A twig blight disease was noticed on bayberry (*Myrica rubra*) trees in Shantou City, Guangdong Province, China, with a high total incidence rate up to 84% in fields in the past several years. After pathogen isolation, purification and pathogenicity tests, at least five fungal species such as *Epicoccum sorghinum*, *Neofusicoccum parvum*, *Lasiodiplodia theobromae*, *Nigrospora oryzae* and a newly classified species *Pestalotiopsis myricae* were confirmed as the pathogens, among them, *P. myricae* is the main pathogen in fields. The results of the indoor fungicide screening revealed that Prochloraz (copper salt) is the most promising fungicide for field disease control, and Pyraclostrobin, 15% Difenoconazole + 15% Propiconazole, Difenoconazole and Myclobutanil could also play a certain role. Moreover, *Bacillus velezensis* strain 3–10 and phytotoxic zeamines from *Dickeya zeae* EC1 could be regarded as potential ecofriendly alternatives in the future.

## Figures and Tables

**Table 1:** Incidence of different bayberry cultivars (4 to 15 years old) in Jinzao town (2018).

Cultivar	Origin	Investigated Trees	Diseased Trees	Incidence (%)
Dongkui	Zhejiang	100	95	95
Fujian Dahong	Fujian	60	48	80
Yingsi	Zhejiang	40	32	80
Wusu	Guangdong	80	12	15
Aodi Wusu	Guangdong	40	12	30
Qingdi	Guangdong	240	62	26
Shanmei	Guangdong	40	13	33

**Table 2:** Fungal isolates and tentative taxonomic categories according to the *ITS*, *tef1-α* and *tub2* sequences.

Taxonomy	Isolate	Orchard	Tree	Cultivar	Tissue
*Colletotrichum gloeosporioides*	C1–C3	Limin	A	Fujian Dahong	Leaf
	C4–C6		B	Yingsi	Leaf
	C7, C8	Caisong Peng	I	Fujian Dahong	Leaf
	C9		II	Yingsi	Leaf
	C10		III	Dongkui	Leaf scar
*Epicoccum sorghinum*	E1	Caisong Peng	II	Yingsi	Petiole base
*Neofusicoccum parvum*	F1, F2	Limin	B	Yingsi	Leaf
	F3				Interior branch
	F4, F5	Caisong Peng	I	Fujian Dahong	Leaf
	F6, F7		III	Dongkui	Leaf scar
	F8				Petiole base
*Lasiodiplodia theobromae*	L1	Caisong Peng	I	Fujian Dahong	Leaf
	L2				Leaf scar
	L3				Interior branch
	L4, L5		II	Yingsi	Interior branch
	L6				Petiole base
*Nigrospora oryzae*	N1	Limin	A	Fujian Dahong	Leaf
	N2	Caisong Peng	II	Yingsi	Leaf
	N3		III	Dongkui	Leaf
	N4, N5				Leaf scar
	N6, N7				Interior branch
	N8, N9				Petiole base
*Pestalotiopsis* sp.	P1, P2	Limin	C	Dongkui	Interior branch
	P3, P4	Caisong Peng	II	Yingsi	leaf
	P5				Interior branch
	P6–P8		III	Dongkui	Leaf scar
	P9				Interior branch
	P10–P14				Leaf scar
*Cytospora tibouchinae*	U1	Limin	B	Yingsi	Interior branch
	V1, V2		C	Dongkui	Interior branch
	V3		D	Qingdi	Interior branch

**Table 3:** The distinctions of morphological characteristics between P4 and other *Pestalotiopsis* spp. in the same phylogenetic branch.

Characteristics	Species					
	*P. myricae*	*P. conigena*	*P. albomaculans*	*P. crassiuscula*	*P. gracilis*	*P. versicolor*
Strain	P4	-	-	Type strain	Type strain	Type strain
Length of conidia (μm)	17.6–26.4	16.9–22.5	15–21.5	20–28	19–23	18–27
Width of conidia (μm)	3.7–6.9	7.3–8.1	6–7	7.5–9.0	6–7	6.5–10
Color of median cells	Concolorous, dark brown to olivaceous	Versicolorous	Versicolorous	Concolorous, brown	Versicolorous	Versicolorous
Length of middle cells (μm)	9.9–17.4	11.7–18.2	12.5–15	14–18	-	12–19
Number of apical appendages	2–4, mostly 2	2–4, mostly 3	3	3	-	2–3, rarely 4
Number of basal appendages	1–2, mostly 1	1	1	1	1	1
Length of apical appendages (μm)	18–40.2	18.2–26.1	10.5–24	10–23	10–26	12–33
Length of basal appendages (μm)	3.1–5.0	3.0–5.2	5.0–8.75	2.5–5.0	-	2–8
Reference	This study	[28]	[27]	[31]	[25]	[26]

**Table 4:** EC_50_ of 16 fungicides against the five identified pathogens.

Fungicide	EC_50_ (mg/L)				
	E1	F7	L3	N9	P4
Pyraclostrobin	0.2738 ± 0.0082	0.0161 ± 0.0018	-	0.0222 ± 0.0007	0.0088 ± 0.0002
Mancozeb	-	-	-	-	-
Prochloraz (copper salt)	1.2396 ± 0.0747	0.0037 ± 0.0004	0.1585 ± 0.0091	0.0004 ± 0.0001	0.0692 ± 0.0022
15% Difenoconazole + 15% Propiconazole	-	0.0456 ± 0.0029	0.4404 ± 0.1073	0.0010 ± 0.0001	-
Iprodione	-	0.2925 ± 0.0135	0.2703 ± 0.0141	-	-
Thiophanate-Methyl	-	-	-	-	-
Tebuconazole	-	0.0528 ± 0.0014	0.3753 ± 0.0139	-	-
Difenoconazole	0.2628 ± 0.0139	0.0460 ± 0.0020	-	0.0132 ± 0.0006	-
Azoxystrobin	-	-	-	0.0001 ± 0.0000	0.0422 ± 0.0007
Chlorothalonil	-	0.3825 ± 0.0077	-		1.8991 ± 0.1375
Myclobutanil	-	0.1320 ± 0.0051	0.6518 ± 0.0481	0.0531 ± 0.0006	-
Matrine	-	-	-	-	-
Boscalid	0.1548 ± 0.0030	-	-	-	-
Hymexazol	-	-	-	-	-
Dithianon	-	-	-	-	-
Carbendazim	-	-	-	-	-

“-” indicates that the value of EC_50_ is not in the range of measured concentration. Values are means (*n* = 3–5) ± SEs.

**Table 5:** Inhibitory activity of EC1 supernatant against the five identified pathogens.

Pathogen	Percentage of EC1 Supernatant in PDA Plate (%)	Percentage of Inhibition (% ± SE)	Results of Multiple Comparisons (α = 0.05)
P4	1	53.5 ± 0.36	c
	5	70.7 ± 0.60	b
	10	94.02 ± 0.37	a
N9	1	71.75 ± 1.6	c
	5	95.26 ± 0.96	b
	10	100.00 ± 0.00	a
F7	1	55.77 ± 1.77	c
	5	86.68 ± 0.66	b
	10	96.64 ± 1.11	a
L3	1	29.22 ± 0.63	c
	5	72.19 ± 2.20	b
	10	88.83 ± 1.63	a
E1	1	31.11 ± 1.38	c
	5	66.58 ± 1.06	b
	10	94.26 ± 0.38	a

Values are means (*n* = 4) ± SEs. Statistical analysis was performed by a one-way analysis of variance (ANOVA). Multiple comparisons were performed by the Least Significant Difference (LSD) method (α = 0.05). Same letters denote non-significant and different letters denote significant differences.

## Supplementary Materials

The following are available online at https://www.mdpi.com/2076-2607/8/5/689/s1.
