# Supplementary material for: Five Fungal Pathogens Are Responsible for Bayberry Twig Blight and Fungicides Were Screened for Disease Control
Source: Microorganisms. 2020 May 8;8(5):689. doi: 10.3390/microorganisms8050689 (PMC7284972; doi:10.3390/microorganisms8050689)
Supplement: Supplementary file 1 [file microorganisms-08-00689-s001.zip › microorganisms-760687-supplementary.docx]

**Supplemental material**

**Five Fungal Pathogens Are Responsible for Bayberry Twig Blight and Fungicides Were Screened for Disease Control**

**Wenjun Li^1,†^, Ming Hu^1,†^, Yang Xue^1^, Zhijun Li^1^, Yanfei Zhang^1^, Daoxu Zheng^2^, Guangtao Lu^3^, Junxia Wang^1,*^, Jianuan Zhou^1,*^**

^1^ Guangdong Laboratory for Lingnan Modern Agriculture, Guangdong Province Key Laboratory of Microbial Signals and Disease Control, Integrative Microbiology Research Center, South China Agricultural University, Guangzhou 510642, China;

^2^ Shantou Forestry Research Institute, Shantou, 515041, China;

^3^ State Key Laboratory for Conservation and Utilization of Subtropical Agro-bioresources, College of Life Science and Technology, Guangxi University, Nanning, China.

† These authors contributed equally to this work.

**Content:**

**Table S1**. Sequences of primers used in this study

**Table S2.** The accession numbers of the *ITS*, *tub2*, *tef1-α*, *LSU* and *SSU* sequences for the five isolates

**Table S3**. Strains used in phylogenetic analyses and their GenBank accession numbers.

**Table S4**. Fungicides used in this study.

**Table S5.** Inhibitory activities of effective fungicides against the five identified pathogens.

**Figure S1.**

**Figure S2**

**Figure S3**

**Figure S4**

**Figure S5**

**Figure S6**

**Figure S7**

**Table S1.** Sequences of primers used in this study

| **Gene** | **Primer name** | **Sequence (5’-3')** | **Reference** |
| --- | --- | --- | --- |
| *ITS* | ITS4 | TCCTCCGCTTATTGATATGC | [16] |
|  | ITS5 | GGAAGTA AAAGTCGTAACAAGG |  |
| *tub2* | BT2A | GGTAACCAAATCGGTGCTGCTTTC | [17] |
|  | BT2B | ACCCTCAGTGTAGTGACCCTTGGC |  |
| *tef1-α* | EF1-526F | GTCGTYGTYAT YGGHCAYGT | [18] |
|  | EF1-1567R | ACHGTRCCRATACCACCRATCTT |  |
| *LSU* | LSU-LR0R | GTACCCGCTGAACTTAAGC | [19] |
|  | LSU-LR7 | TACTACCACCAAGATCT |  |
| *SSU* | SSU-NS1 | GTAGTCATATGCTTGTCTC | [19] |
|  | SSU-NS4 | CTTCCGTCAATTCCTTTAAG |  |

**Table S2.** The accession numbers of the *ITS*, *tub2*, *tef1-α*, *LSU* and *SSU* sequences for the five isolates

| Isolate | GenBank Accessions | | | | |
| --- | --- | --- | --- | --- | --- |
|  | *ITS* | *tub2* | *tef1-α* | *LSU* | *SSU* |
| E1 | MN180878 | MN461163 | MN461167 | MN181369 | MN180884 |
| F7 | MN180877 | MN461162 | MN461166 | MN181368 | MN180883 |
| L3 | MN180881 | MN461165 | MN461169 | MN181372 | MN180887 |
| N9 | MN180880 | MN461170 | MN507730 | MN181371 | MN180886 |
| P4 | MN180879 | MN461164 | MN461168 | MN181370 | MN180885 |

**Table S3.** Strains used in phylogenetic analyses and their GenBank accession numbers.

| **GenBank Accessions** | | **Species** | **Strain** |
| --- | --- | --- | --- |
| ***ITS*** | ***tub2*** |  |  |
| MN180878 | MN461163 | *Epicoccum sorghinum* | E1 |
| KT310092 | MH249627 |  | USPMTOX48^T^ |
| MK516206 | MK516208 |  | GZDS2018BXT010^T^ |
| FJ427067 | FJ427173 |  | CBS 179.80 |
| GU237795 | GU237607 | *Epicoccum draconis* | CBS 186.83 |
| FJ426995 | FJ427106 | *Epicoccum nigrum* | CBS 125.82 |
| GU014950 | GU563404 |  | CBS 161.73 |
| GU237760 | GU237588 | *Epicoccum brasiliense* | CBS 120105^T^ |
| KY742091 | KY742333 | *Epicoccum camelliae* | LC:4858^T^ |
| KY742092 | KY742334 |  | LC:4862 |
| KY742093 | KY742335 | *Epicoccum dendrobii* | LC:8145^T^ |
| KY742095 | KY742337 | *Epicoccum duchesneae* | LC:5139^T^ |
| GU237731 | GU237612 | *Epicoccum henningsii* | CBS 104.80^T^ |
| KY742097 | KY742339 | *Epicoccum hordei* | LC:8148^T^ |
| GU237732 | GU237615 | *Epicoccum huancayense* | CBS 105.80^T^ |
| KY742099 | KY742341 | *Epicoccum italicum* | LC:8150^T^ |
| KY742101 | KY742343 | *Epicoccum latusicollum* | LC:5158^T^ |
| KY742102 | KY742344 |  | LC:4859 |
| KY742107 | KY742349 | *Epicoccum layuense* | LC:8155^T^ |
| FJ427049 | FJ427159. | *Epicoccum pimprinum* | CBS 246.60^T^ |
| MH861377 | GU237647 | *Epicoccum plurivorum* | CBS 558.8^T^ |
| GU237822 | GU237646 |  | CBS 284.93 |
| KT310008 | KT309586 |  | ICMP:6873T |
| KY742113 | KY742355 | *Epicoccum poae* | LC:8160^T^ |
| MK100177 | MK140527 |  | R2I2 |
| KY742118 | KY742360 | *Epicoccum viticis* | LC:5126^T^ |
| KY742117 | KY742359 |  | BRIP:29294 |
| JF740201 | KT389804 | *Leptosphaeria conoidea* | CBS 616.75 |
| MN180877 | MN461162 | *Neofusicoccum parvum* | F7 |
| KF766204 | AY236917 |  | CMW9081^T^ |
| AY259098 | EU673095 |  | CBS110301 |
| MG836707 | MG879024 |  | LW43-2 |
| MG836708 | MG879025 |  | LW45-1 |
| KU997474 | KU997593 |  | MAR2134 |
| GU251155 | GU251815 | *Neofusicoccum andinum* | PD252 |
| KT728917 | KT728913 | *Neofusicoccum batangarum* | CMM4553 |
| KT728916 p | CMM4547 |  | CMM4547 |
| MG645054 | MG649076 | *Neofusicoccum occulatum* | C |
| MH864743 | EU339472 |  | CBS 128008^T^ |
| KF766206 | KF766145 | *Neofusicoccum umdonicola* | CBS 123645^T^ |
| EU339525 | EU339477 | *Neofusicoccum ribis* | MUCC125 |
| EU375516 | EU375520 | *Neofusicoccum australe* | JL619 |
| HQ529747 | HQ529717 |  | UCR748 |
| KP860869 | KP860789 | *Neofusicoccum luteum* | CMW42348 |
| HQ529764 | HQ529773 |  | UCR444 |
| GU251152 | GU251812 | *Fusicoccum arbuti* | PD282 |
| EU821903 | EU821843 | *Neofusicoccum cordaticola* | CMW14056 |
| KY052992 | KY000164 |  | MAR24 |
| KX464168 | KX464957 | *Neofusicoccum corticosae* | CBS 118099 |
| NR_152945 | AY615173 | *Neofusicoccum mangiferae* | CMW 7797^T^ |
| KY052921 | KY000107 |  | MAN19 |
| GU251156 | GU251816 | *Neofusicoccum nonquaesitum* | PD86 |
| GU251157 | GU251817 |  | PD90 |
| EF591925 | EF591959 | *Neofusicoccum pennatisporum* | MUCC510^T^ |
| EF591912 | EF591948 | *Neofusicoccum protearum* | MUCC497 |
| KX464228 | KX465060 | *Neofusicoccum viticlavatum* | CBS 123532 |
| JX515724 | JX515692 | *Neofusicoccum vitifusiforme* | UCD622-Oe |
| KC706914 | KC706922 | *Neofusicoccum macroclavatum* | NZM353 |
| DQ093196 | DQ093206. |  | WAC12444 |
| KX306953 | KT581221 | *Botryosphaeria dothidea* | IOT B006 |
| DQ233600 | DQ233621 |  | UCD1064So |
| MN180881 | MN461165 | *Lasiodiplodia theobromae* | L3 |
| NR_111174 | KU887532 |  | CBS 164.96^T^ |
| FJ150695 | KU887531 |  | CBS 111530 |
| LC270866 | LC314725 | *Lasiodiplodia pseudotheobromae* | 17-004 |
| LC270865 | LC314724 |  | 17-003 |
| KT852959 | KU887520 | *Lasiodiplodia margaritacea* | CBS 122519^T^ |
| KY052994 | KY000166 | *Lasiodiplodia egyptiacae* | MAR27 |
| KY052995 | KY000167 |  | MAR29 |
| NR_147329 | KP872413 | *Lasiodiplodia hormozganensis* | IRAN 1500Cl^T^ |
| GU945356 | KP872414 |  | IRAN1498C |
| MG954349 | MG979547 |  | CMM 4620 |
| DQ458892 | DQ458860 | *Lasiodiplodia gonubiensis* | CBS115812^T^ |
| KU887124 | KU887502 |  | CMW36240 |
| DQ103552 | KP872407 | *Lasiodiplodia crassispora* | CMW13488 |
| GU799455 | GU799478 |  | UCD23Co |
| KF766194 | KU887534 | *Lasiodiplodia venezuelensis* | CMW 13512 |
| JX545100 | JX545140 |  | CBS 129753 |
| MH861166 | KP872419 | *Lasiodiplodia parva* | CBS 456.78^T^ |
| GU945354 | KP872406 | *Lasiodiplodia citricola* | IRAN1522C^T^ |
| GU945353 | KP872405 |  | IRAN1521C |
| GU945351 | KP872411 | *Lasiodiplodia gilanensis* | IRAN1523C^T^ |
| GU945352 | KP872412 |  | IRAN1501C |
| GU945347 L | KP872416 | *asiodiplodia iraniensis* | IRAN1502C |
| GU945348 | KP872415 |  | IRAN1520C^T^ |
| KP699095 | KP699091 | *Lasiodiplodia viticola* | LAG05 |
| HQ288228 | HQ288307 |  | UCD2604MOe^T^ |
| AY259091 | DQ458848 | *Botryosphaeria lutea* | CBS110299 |
| AY236946 | AY339249 |  | CMW9076 |
| MN180879 | MN461164 | *Pestalotiopsis myricae* | P4 |
| HM535722.1 | HM573257 | *Pestalotiopsis gracilis* | LL-TEZ |
| HM535706 | HM573242 |  | HHL-FG |
| HM535733 | HM573268 | *Pestalotiopsis conigena* | QQ-AZ |
| HM535700.1 | HM573237 | *Pestalotiopsis zonata* | BGR-AY |
| DQ813435 | DQ787841 | *Pestalotiopsis virgatula* | PSHI2004Endo415 |
| HM535711 | HM573247 | *Pestalotiopsis crassiuscula* | HMG-TAZ |
| DQ789378 | DQ657886 | *Pestalotiopsis albomaculans* | PSHI2004Endo472 |
| JN861777 | JN861772 | *Pestalotiopsis microspora* | YS44 |
| JN861776 | JN861771.2 |  | YS26 |
| JN314418 | JN314419 |  | RA1-2 |
| KR703275 | KU377338 |  | KFRD-2 |
| JN861775 | JN861770 | *Pestalotiopsis versicolor* | RA2-1 |
| JN861774.2 | JN861769 |  | XJ42 |
| JN861773 | JN861768 |  | XJ27 |
| DQ334862 | DQ333585 |  | PSHI2004Endo124 |
| JX399006 | JX399037 | *Pestalotiopsis adusta* | ICMP 6088^T^ |
| JX399007 | JX399038 |  | MFLUCC 10-146 |
| KC247154 | KC247155 | *Pestalotiopsis anacardiacearum* | IFRDCC 2397^T^ |
| KM199341 | KM199427 | *Pestalotiopsis arceuthobii* | CBS 434.65^T^ |
| KM199340 | KM199426 | *Pestalotiopsis arengae* | CBS 331.92^T^ |
| KM199297 | KM199409 | *Pestalotiopsis australasiae* | CBS 114126^T^ |
| KM199332 | KM199383 | *Pestalotiopsis australis* | CBS 114193^T^ |
| KM199308 | KM199399 | *Pestalotiopsis biciliata* | CBS 124463^T^ |
| KM199309 | KM199401 |  | CBS 236.38 |
| JX399010 | JX399041 | *Pestalotiopsis camelliae* | MFLUCC 12-0277^T^ |
| JX399011 | JX399042 |  | MFLUCC 12-0278 |
| JX398990 | JX399025 | *Pestalotiopsis clavata* | MFLUCC 12-0268^T^ |
| KM199314 | KM199416 | *Pestalotiopsis diploclisia* | CBS 115449 |
| KM199320 | KM199419 |  | CBS 115587^T^ |
| JX399009 | JX399040 | *Pestalotiopsis diversiseta* | MFLUCC 12-0287^T^ |
| KC537805 | KC537819 | *Pestalotiopsis gaultheria* | IFRD 411-014^T^ |
| KM199300 | KM199407 | *Pestalotiopsis grevilleae* | CBS 114127^T^ |
| KM199302 | KM199395 | *Pestalotiopsis kenyana* | CBS 442.67^T^ |
| KM199303 | KM199396 |  | CBS 911.96 |
| JX398992 | JX399027 | *Pestalotiopsis linearis* | MFLUCC 12-0271^T^ |
| KM199312 | KM199404 | *Pestalotiopsis parva* | CBS 265.37^T^ |
| JX398999 | JX399030 | *Pestalotiopsis unicolor* | MFLUCC 12-0276^T^ |
| JQ683725 | JQ683709 | *Seiridium sp.* | SD096 |
| ***ITS*** | ***LSU*** | **Species** | **Strain** |
| MN180880 | MN181371 | *Nigrospora oryzae* | N9 |
| KX958066 | KX958066 |  | 17R-9-F01 |
| KX985944 | KX986101 |  | LC2693 |
| MH443350 | MH443370 |  | ERL2-10 |
| KX985993 | KY806253 | *Nigrospora sphaerica* | LC4264 |
| KX986010 | KX986106 | *Nigrospora osmanthi* | CGMCC38126^T^ |
| KX985986 | KX986103 | *Nigrospora camelliae-sinensis* | CGMCC38125^T^ |
| KX986064 | KX986098 | *Nigrospora aurantiaca* | CGMCC38130^T^ |
| KX986023 | KX986107 | *Nigrospora chinensis* | CGMCC38127^T^ |
| KX986048 | KX986109 | *Nigrospora gorlenkoana* | CBS 480.73^T^ |
| KX985983 | KX986113 | *Nigrospora guilinensis* | CGMCC38124^T^ |
| KX986091 | KX986112 | *Nigrospora hainanensis* | CGMCC38129^T^ |
| KX985978 | KX98610 | *Nigrospora lacticolonia* | CGMCC38123^T^ |
| KX986076 | KX986110 | *Nigrospora musae* | CBS 319.34^T^ |
| GU017506 | GU017547 | *Nigrospora sp.* | KH00291 |
| KF144896 | KF144942 | *Arthrinium malaysianum* | CBS 102053 |

Superscript T indicates type/holotype strain, and superscript R denotes representative strain. In case sequences from type strains were not available, those from published paper were included.

**Table S4**. Fungicides used in this study

| **Product names** | **Active ingredients** | **Manufacturer** | **Concentration** | **Recommended concentration for field application (mg/L)** |
| --- | --- | --- | --- | --- |
| Pyraclostrobin | Pyraclostrobin | Huifeng Agrochemical, China | 250 g/L | 250 |
| 80% Mancozeb | Mancozeb | Limin Chemical, China | 80% | 1600 |
| Shibaike Jin | Prochloraz (copper salt) | Huifeng Agrochemical, China | 50% | 425.45 |
| Difenoconazole + Propiconazole | 15% Difenoconazole + 15% Propiconazole | Hulian, China | 30% | 96.3 |
| ROVRAL | Iprodione | FMC, China | 500 g/L | 500 |
| Abe | Thiophanate-Methyl | Brightmart, China | 80% | 1360 |
| Tebuconazole | Tebuconazole | Hulian, China | 80% | 160 |
| Kaimeilai | Difenoconazole | KYX Chemical, China | 60% | 420 |
| Fenkang | Azoxystrobin | KYX Chemical, China | 25% | 283.5 |
| Limin Tonglin | Chlorothalonil | Limin Chemical, China | 75% | 1875 |
| Myclobutanil | Myclobutanil | Yifan Biotech, China | 25% | 338 |
| Baifabaizhong | Matrine | Tangpule, China | 0.5% | 6.76 |
| CANTUS | Boscalid | BASF, Germany | 50 % | 150 |
| Hymexazol | Hymexazol | Heyi Chemicals, China | 30% | 616.23 |
| Boqing | Dithianon | Heyi Chemicals, China | 22.7% | 301.1836 |
| Carbendazim | Carbendazim | Huayou, China | 100% | 10000 |

**Table S5.** Inhibitory activities of effective fungicides against the five identified pathogens.

| **Pathogen** | **Fungicide** | Concentration of Pesticide (mg/L) | Inhibition rate **(% ± SE)** | **Results of multiple comparisons (α = 0.05)** |
| --- | --- | --- | --- | --- |
| E1 | Pyraclostrobin | 0.0025 | 1.52 ± 0.87 | d |
|  |  | 0.025 | 32.03 ± 0.83 | c |
|  |  | 0.25 | 45.45 ± 0.87 | b |
|  |  | 2.5 | 72.29 ± 0.71 | a |
|  | Prochloraz  (copper salt) | 0.00425 | 13.85 ± 1.92 | d |
|  |  | 0.0425 | 17.75 ± 1.12 | c |
|  |  | 0.425 | 38.96 ± 0.43 | b |
|  |  | 4.25 | 63.64 ± 0.71 | a |
|  | Difenoconazole | 0.0042 | 6.06 ± 0.43 | d |
|  |  | 0.042 | 25.97 ± 0.50 | c |
|  |  | 0.42 | 62.77 ± 0.50 | b |
|  |  | 4.2 | 76.19 ± 1.32 | a |
|  | Boscalid | 0.0015 | 0.00 | e |
|  |  | 0.0075 | 6.06 ± 1.78 | d |
|  |  | 0.015 | 12.59 ± 0.36 | c |
|  |  | 0.15 | 51.95 ± 0.43 | b |
|  |  | 1.5 | 100.00 | a |
| F7 | Pyraclostrobin | 0.00025 | 8.33 ± 2.04 | f |
|  |  | 0.00125 | 38.19 ± 2.81 | e |
|  |  | 0.0025 | 43.90 ± 1.05 | d |
|  |  | 0.025 | 51.63 ± 0.78 | c |
|  |  | 0.25 | 63.01 ± 1.80 | b |
|  |  | 2.5 | 92.68 ± 1.41 | a |
|  | Prochloraz  (copper salt) | 0.000425 | 2.08 ± 1.04 | f |
|  |  | 0.00085 | 57.44 ± 1.16 | e |
|  |  | 0.00425 | 62.60 ± 2.23 | d |
|  |  | 0.0425 | 73.17 ± 0.91 | c |
|  |  | 0.425 | 84.55 ± 0.91 | b |
|  |  | 4.25 | 100.00 | a |
|  | 15% Difenoconazole + 15% Propiconazole | 0.000963 | 13.17 ± 0.04 | e |
|  |  | 0.00963 | 21.63 ± 0.61 | d |
|  |  | 0.04815 | 50.54 ± 1.66 | c |
|  |  | 0.0963 | 58.7 ± 1.83 | b |
|  |  | 0.963 | 88.62 ± 0.51 | a |
|  | Iprodione | 0.005 | 1.63 ± 1.00 | d |
|  |  | 0.05 | 12.60 ± 0.78 | c |
|  |  | 0.5 | 50.41 ± 1.94 | b |
|  |  | 5 | 100.00 | a |
|  | Tebuconazole | 0.0016 | 2.44 ± 0.89 | e |
|  |  | 0.016 | 31.71 ± 1.36 | d |
|  |  | 0.08 | 41.22 ± 1.19 | c |
|  |  | 0.16 | 78.21 ± 1.90 | b |
|  |  | 1.6 | 100.00 | a |
|  | Difenoconazole | 0.0042 | 15.05 ± 2.38 | e |
|  |  | 0.021 | 37.28 ± 1.70 | d |
|  |  | 0.042 | 52.33 ± 1.08 | c |
|  |  | 0.42 | 89.92 ± 0.61 | b |
|  |  | 4.2 | 100.00 | a |
|  | Chlorothalonil | 0.009375 | 6.09 ± 0.72 | e |
|  |  | 0.01875 | 16.42 ± 0.65 | d |
|  |  | 0.1875 | 41.79 ± 0.61 | c |
|  |  | 1.875 | 72.68 ± 0.95 | b |
|  |  | 18.75 | 90.89 ± 0.40 | a |
|  | Myclobutanil | 0.00338 | 6.82 ± 0.55 | d |
|  |  | 0.0338 | 23.39 ± 0.99 | c |
|  |  | 0.338 | 58.37 ± 1.22 | b |
|  |  | 3.38 | 100.00 | a |
| L3 | Prochloraz  (copper salt) | 0.00425 | 23.69 ± 0.42 | c |
|  |  | 0.0425 | 44.77 ± 1.99 | b |
|  |  | 0.425 | 62.48 ± 0.42 | a |
|  |  | 4.25 | 65.01 ± 0.81 | a |
|  | 15% Difenoconazole + 15% Propiconazole | 0.000963 | 10.62 | d |
|  |  | 0.00963 | 22.85 ± 0.42 | c |
|  |  | 0.0963 | 31.70 ± 1.76 | b |
|  |  | 0.963 | 62.06 ± 1.61 | a |
|  | Iprodione | 0.005 | 5.56 ± 1.19 | d |
|  |  | 0.05 | 11.47 ± 2.12 | c |
|  |  | 0.5 | 61.21 | b |
|  |  | 5 | 92.83 ± 0.81 | a |
|  | Tebuconazole | 0.0016 | 4.30 ± 1.26 | d |
|  |  | 0.016 | 12.73 ± 1.26 | c |
|  |  | 0.16 | 36.76 ± 1.46 | b |
|  |  | 1.6 | 69.64 | a |
|  | Myclobutanil | 0.00338 | 7.67 ± 2.12 | d |
|  |  | 0.0338 | 20.74 ± 1.95 | c |
|  |  | 0.338 | 38.45 ± 1.69 | b |
|  |  | 3.38 | 69.65 ± 2.38 | a |
| N9 | Pyraclostrobin | 0.000125 | 5.05 ± 1.01 | d |
|  |  | 0.00025 | 18.18 | c |
|  |  | 0.0025 | 41.41 ± 3.94 | b |
|  |  | 0.025 | 46.46 ± 1.01 | b |
|  |  | 0.25 | 77.27 ± 1.52 | a |
|  |  | 2.5 | 77.78 ± 1.82 | a |
|  | Prochloraz  (copper salt) | 0.000425 | 50.38 ± 1.31 | c |
|  |  | 0.00425 | 67.80 ± 3.35 | b |
|  |  | 0.0425 | 72.72 ± 2.55 | b |
|  |  | 0.425 | 100.00 | a |
|  |  | 4.25 | 100.00 | a |
|  | 15% Difenoconazole + 15% Propiconazole | 0.00004815 | 13.46 ± 2.12 | f |
|  |  | 0.0000963 | 46.92 ± 0.44 | e |
|  |  | 0.0004815 | 52.69 ± 1.46 | d |
|  |  | 0.00963 | 64.77 ± 0.73 | c |
|  |  | 0.0963 | 74.24 | b |
|  |  | 0.963 | 100.00 | a |
|  | Difenoconazole | 0.00021 | 1.15 ± 0.74 | g |
|  |  | 0.00042 | 7.31 ± 0.97 | f |
|  |  | 0.0021 | 12.69 ± 0.74 | e |
|  |  | 0.0042 | 62.88 ± 0.76 | d |
|  |  | 0.042 | 79.17 ± 1.79 | c |
|  |  | 0.42 | 85.23 ± 0.73 | b |
|  |  | 4.2 | 100.00 | a |
|  | Azoxystrobin | 0.00014175 | 41.15 ± 0.74 | f |
|  |  | 0.0002835 | 56.54 ± 0.38 | e |
|  |  | 0.0014175 | 63.46 ± 0.74 | d |
|  |  | 0.002835 | 73.48 ± 0.76 | c |
|  |  | 0.2835 | 81.82 | b |
|  |  | 2.835 | 89.39 ± 1.75 | a |
|  | Myclobutanil | 0.00338 | 10.38 ± 0.38 | e |
|  |  | 0.0338 | 46.15 | d |
|  |  | 0.169 | 61.15 ± 0.38 | c |
|  |  | 0.338 | 84.85 ± 0.87 | b |
|  |  | 3.38 | 100.00 | a |
| P4 | Pyraclostrobin | 0.00025 | 2.03 | f |
|  |  | 0.00125 | 33.28 ± 0.49 | e |
|  |  | 0.0025 | 37.19 | d |
|  |  | 0.025 | 66.53 ± 1.70 | c |
|  |  | 0.25 | 96.28 ± 1.04 | b |
|  |  | 2.5 | 100 | a |
|  | Prochloraz  (copper salt) | 0.000425 | 19.76 ± 0.84 | e |
|  |  | 0.00425 | 23.97 | d |
|  |  | 0.0425 | 30.99 ± 0.41 | c |
|  |  | 0.425 | 63.63 | b |
|  |  | 4.25 | 92.15 ± 0.41 | a |
|  | Azoxystrobin | 0.002835 | 16.94 ± 1.24 | e |
|  |  | 0.014175 | 35.39 ± 0.42 | d |
|  |  | 0.02835 | 47.93 ± 1.07 | c |
|  |  | 0.2835 | 81.82 | b |
|  |  | 2.835 | 89.26 ± 0.48 | a |
|  | Chlorothalonil | 0.009375 | 1.60 ± 0.81 | d |
|  |  | 0.01875 | 22.31 | c |
|  |  | 0.1875 | 23.97 ± 0.67 | c |
|  |  | 1.875 | 45.04 ± 1.04 | b |
|  |  | 18.75 | 74.38 ± 1.07 | a |

Values are means (n =3～5) ± SEs. Statistical analysis was performed by one-way analysis of variance (ANOVA). Multiple comparison was performed by Least Significant Difference (LSD) method (α=0.05). Same letters denote non-significant and different letters denote significant differences.


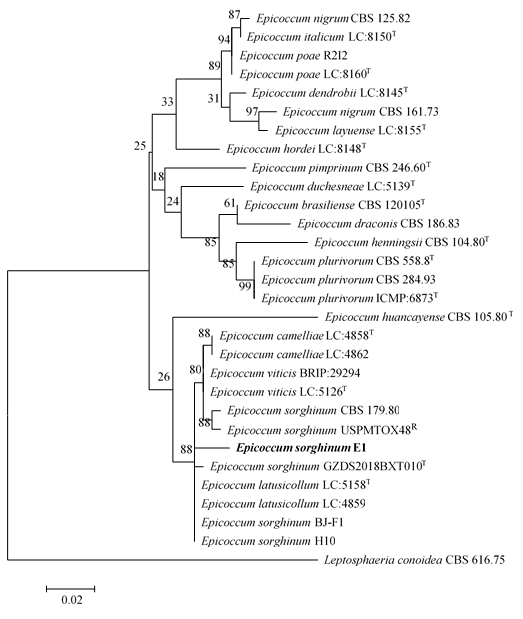


**Figure S1. Phylogenetic tree of isolate E1 based on the combination of *ITS* and *tub2* sequences from distinctive species of *Epicoccum*.** Joint *ITS*+*tub2* sequence of *Leptosphaeria conoidea* strain CBS 616.75 was used as an outgroup. The Maximum Likelihood method in the software MEGA 6.0 was used to construct the tree. The number at each branch indicated the bootstrap value in 1,000 replications. Superscript T denotes type strain, and superscript R denotes representative strain.

**
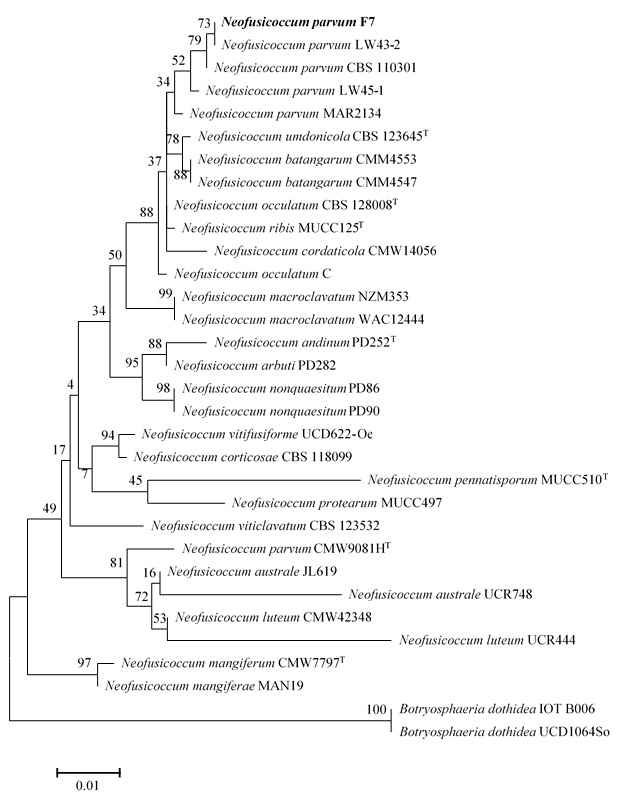
**

**Figure S2. Phylogenetic tree of isolate F7 based on the combination of *ITS* and *tub2* sequences from distinctive species of *Neofusicoccum*.** *ITS* and *tub2* sequences from two *Botryosphaeria dothidea* strains were used as outgroups. The Maximum Likelihood method in the software MEGA 6.0 was used to construct the tree. The number at each branch indicated the bootstrap value in 1,000 replications. Superscript T denotes type strain.

**
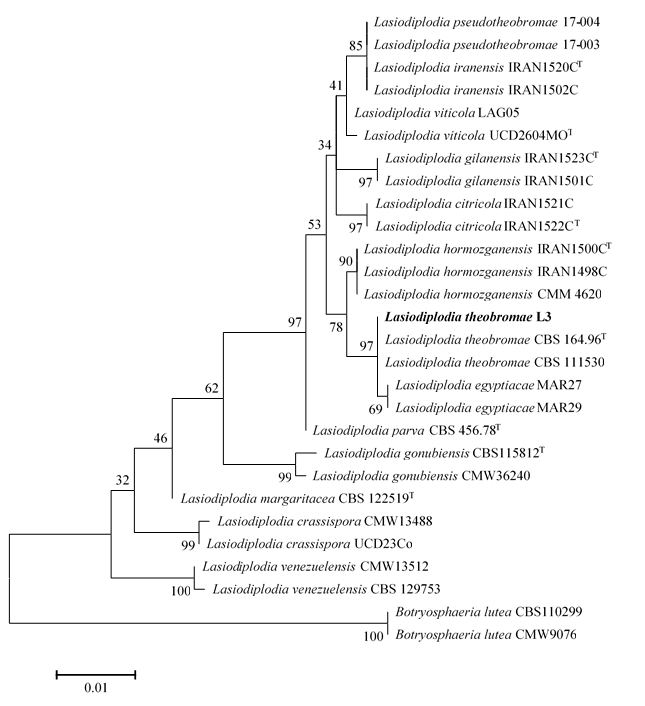
**

**Figure S3. Phylogenetic tree of isolate L3 based on the combination of *ITS* and *tub2* sequences from distinctive species of *Lasiodiplodia*.** *ITS* and *tub2* sequences from two *Botryosphaeria lutea* strains were used as outgroups. The Maximum Likelihood method in the software MEGA 6.0 was used to construct the tree. The number at each branch indicated the bootstrap value in 1,000 replications. Superscript T denotes type strain.

**B**


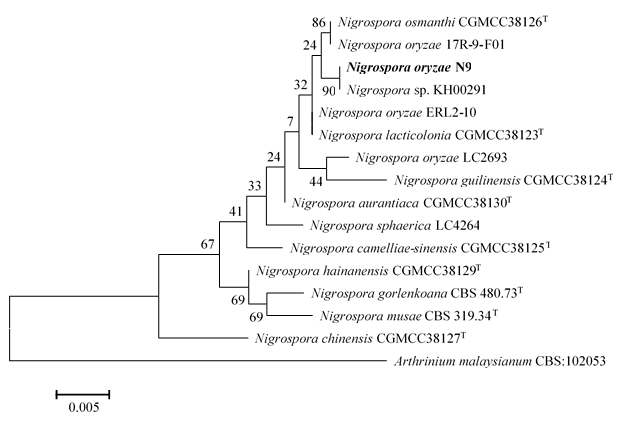


**Figure S4. Phylogenetic trees of isolate N9 based on the combination of *ITS* and *LSU* sequences from distinctive species of *Nigrospora*.** Combination of *ITS* and *LSU* sequences from *Arthrinium malaysianum* strain CBS:102053 were used as an outgroup. The Maximum Likelihood method in the software MEGA 6.0 was used to construct the tree. The number at each branch indicated the bootstrap value in 1,000 replications. Superscript T denotes type strain.


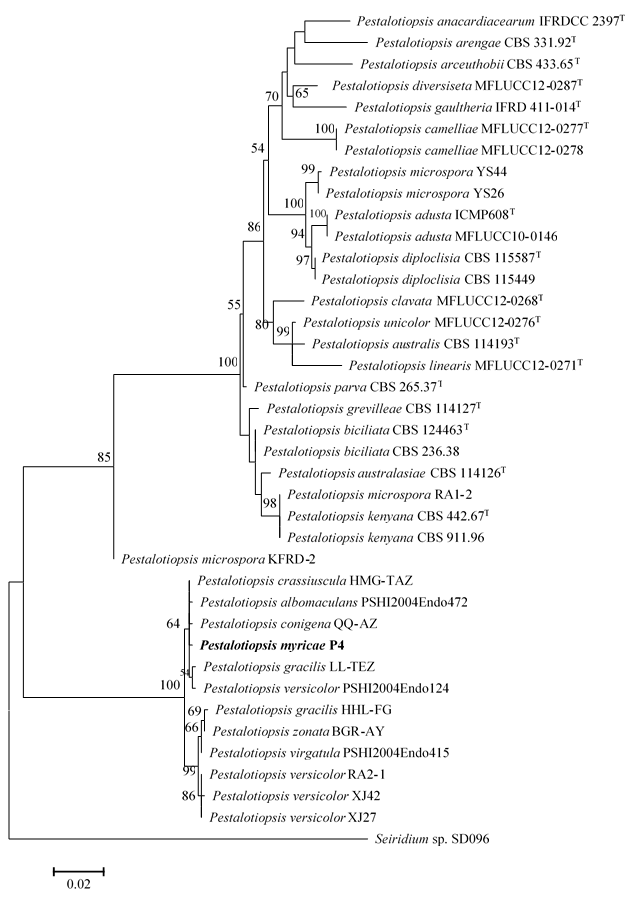


**Figure S5. Phylogenetic tree of isolate P4 based on the combination of *ITS* and *tub2* sequences from distinctive species of *Pestalotiopsis*.** Combination of *ITS* and *tub2* sequences from *Seiridium* strain was used as an outgroup. The Maximum Likelihood method in the software MEGA 6.0 was used to construct the tree. The number at each branch indicated the bootstrap value in 1,000 replications. Superscript T denotes type strain. Hide frequency values lower than 50%.

**A**


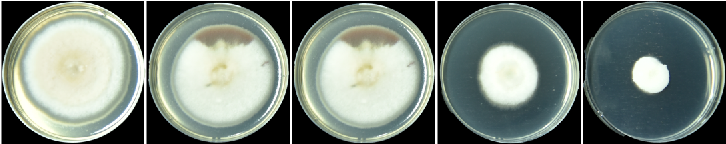

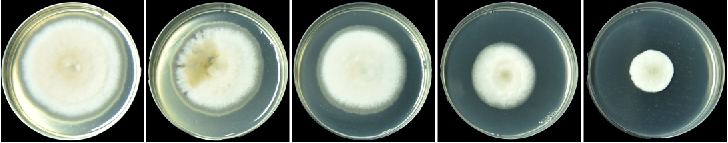

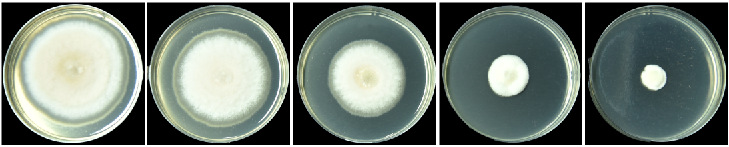

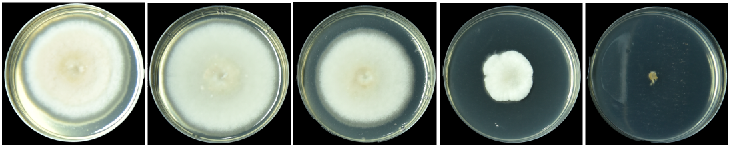


**CK 10^-5^ 10^-4^ 10^-3^ 10^-2^**

Pyraclostrobin

Prochloraz (copper salt)

Difenoconazole

Boscalid

**B**

**
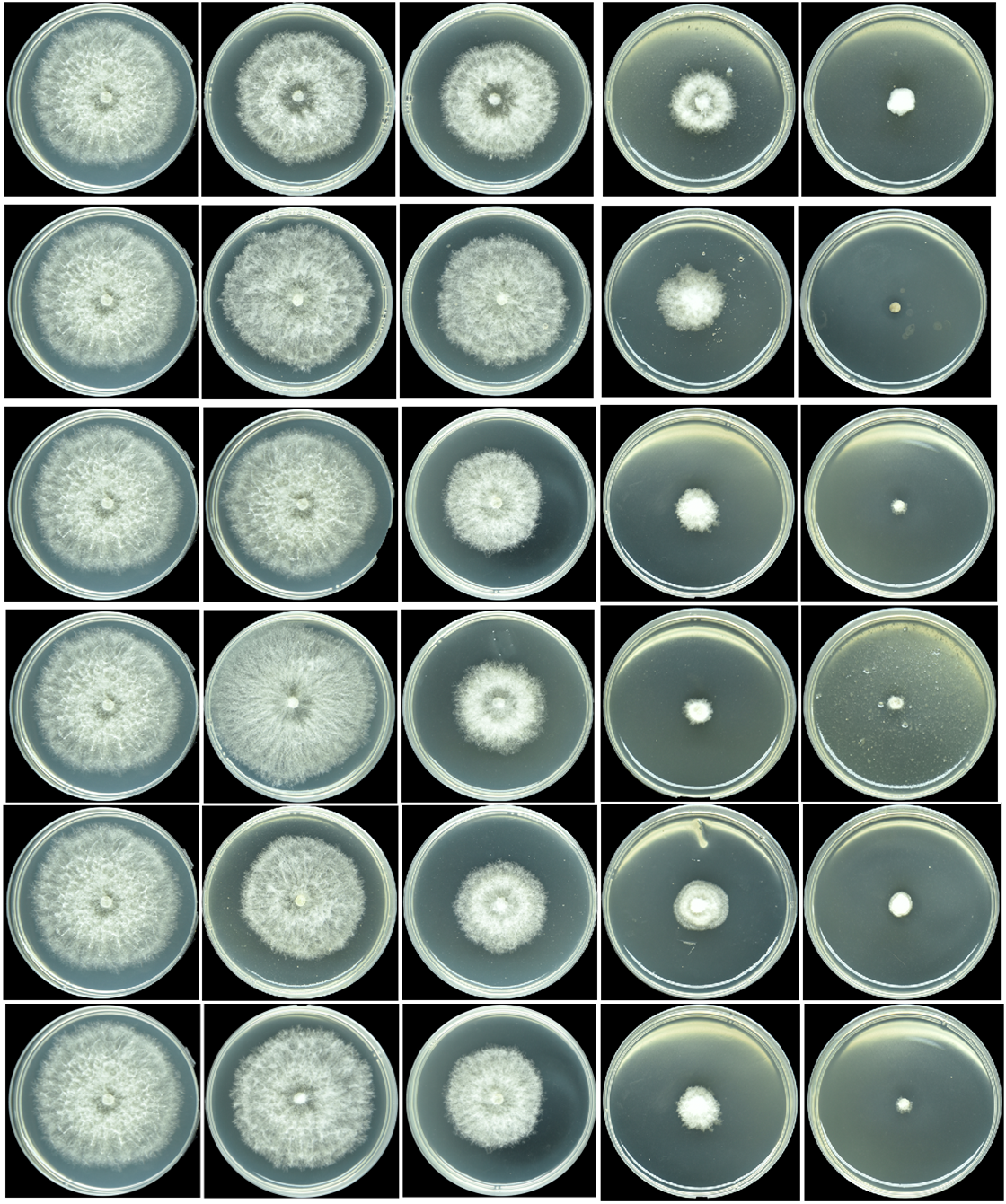

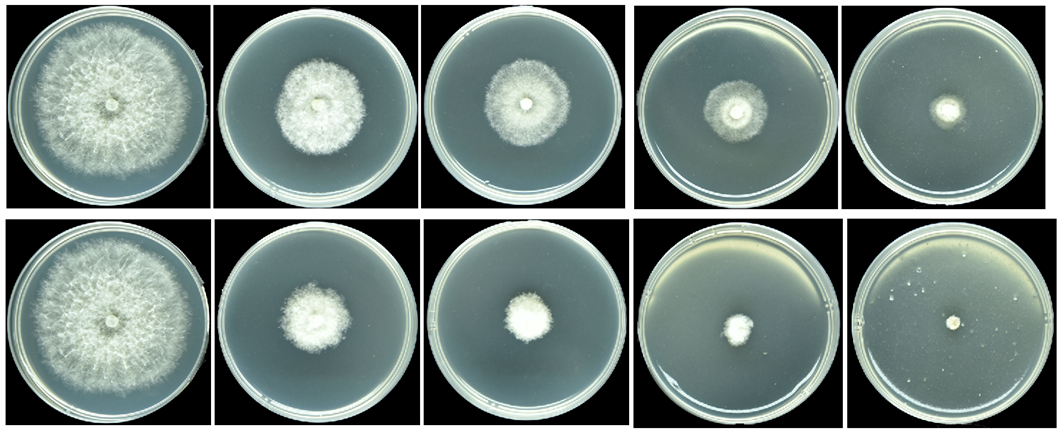
**

Prochloraz (The copper salt)

Pyraclostrobin

Myclobutanil

Chlorothalonil

Difenoconazole

Tebuconazole

15% Difenoconazole + 15% Propiconazole

Iprodione

**CK 10^-5^ 10^-4^ 10^-3^ 10^-2^**

**C**


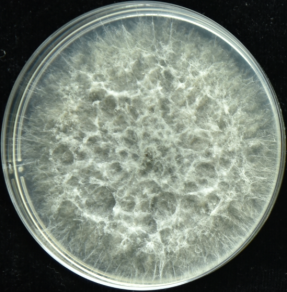


**CK 10^-5^ 10^-4^ 10^-3^ 10^-2^**


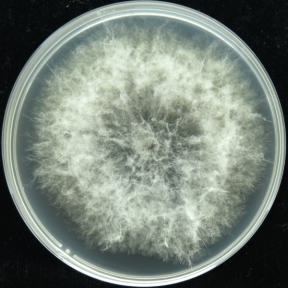

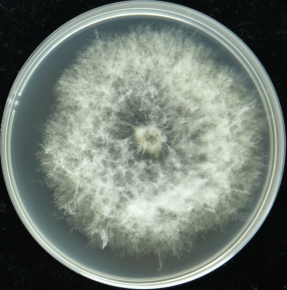

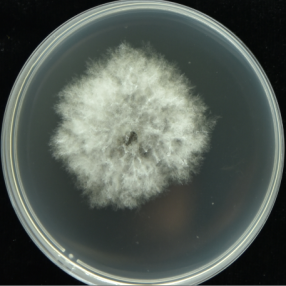

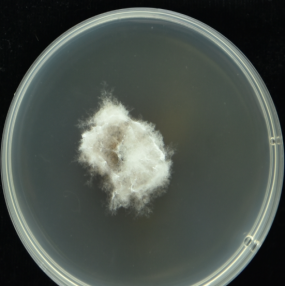

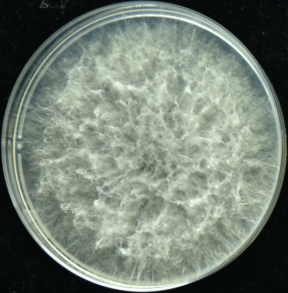

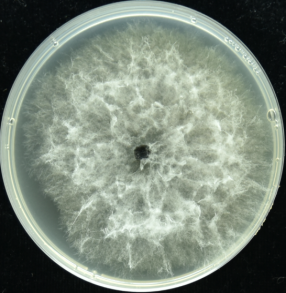

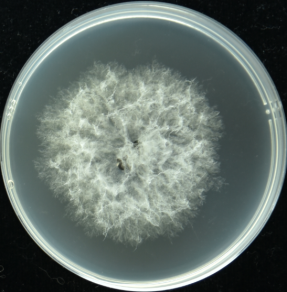

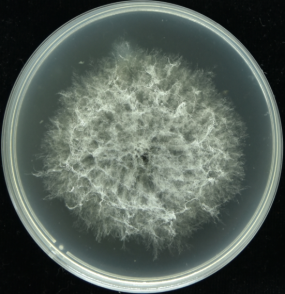

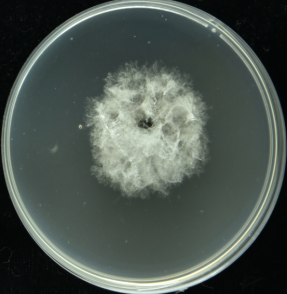

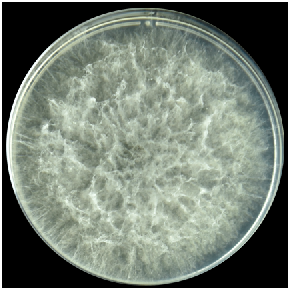

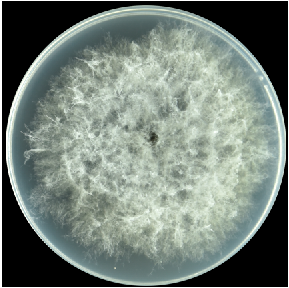

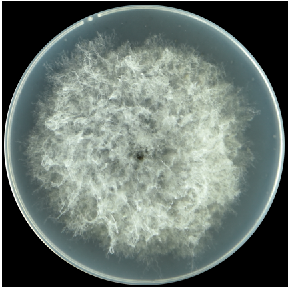

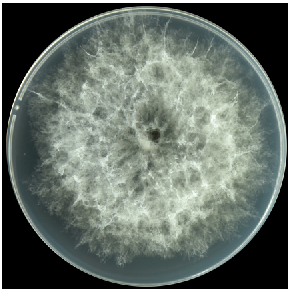

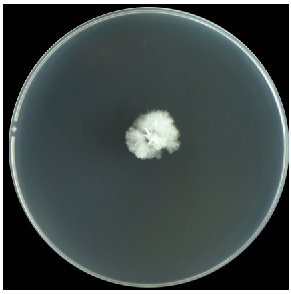

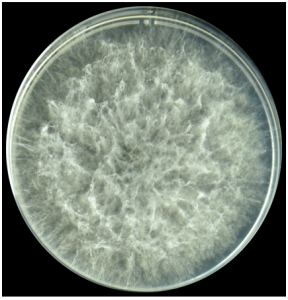

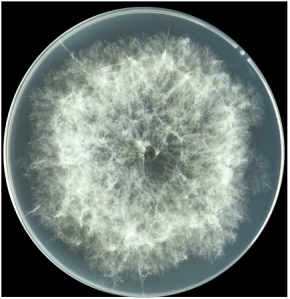

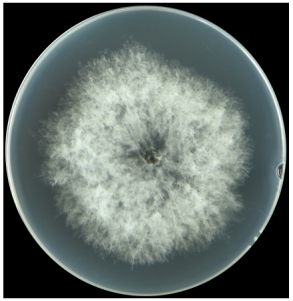

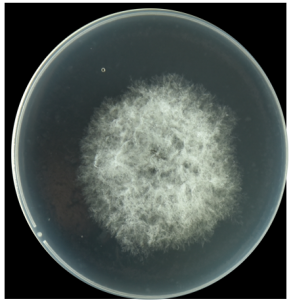

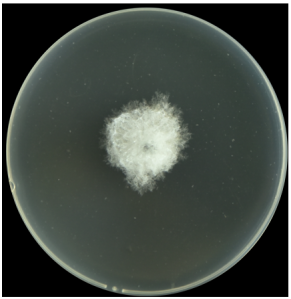

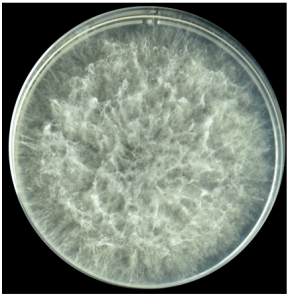

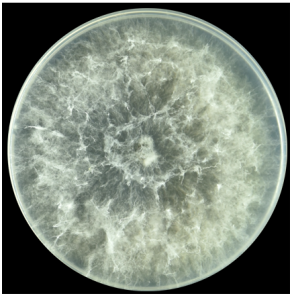

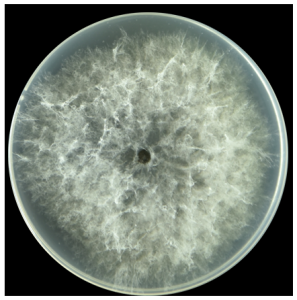

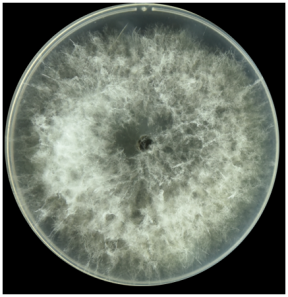

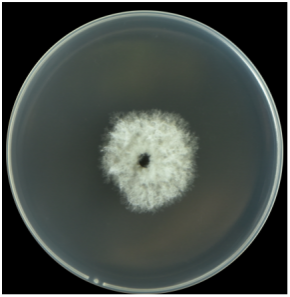


Myclobutanil

Prochloraz (copper salt)

15% Difenoconazole + 15% Propiconazole

Iprodione

Tebuconazole

**D**


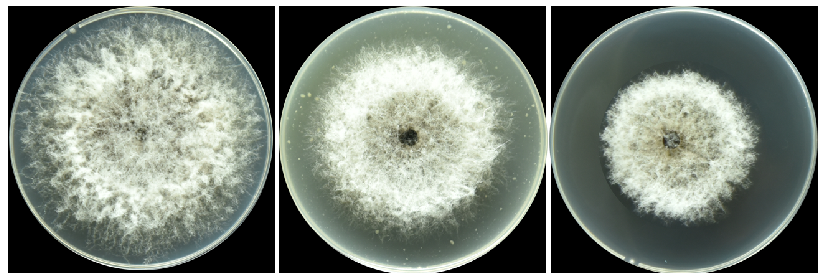

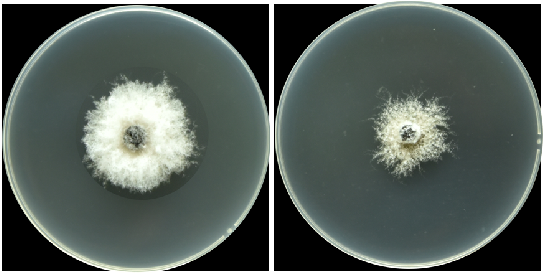


Pyraclostrobin


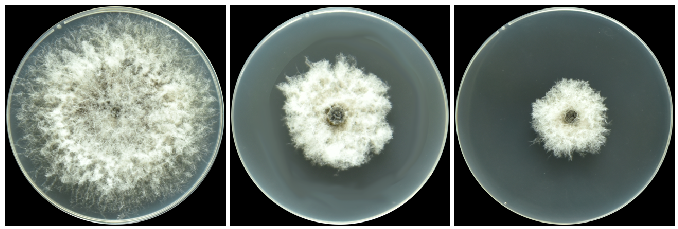

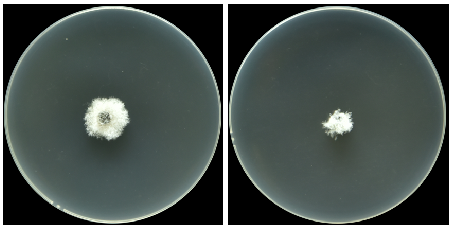


Prochloraz (copper salt)


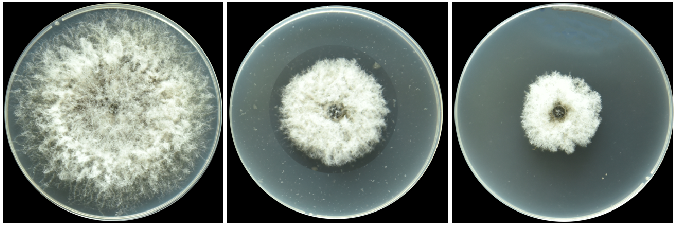

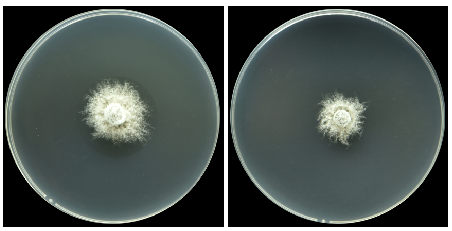


15% Difenoconazole + 15% Propiconazole


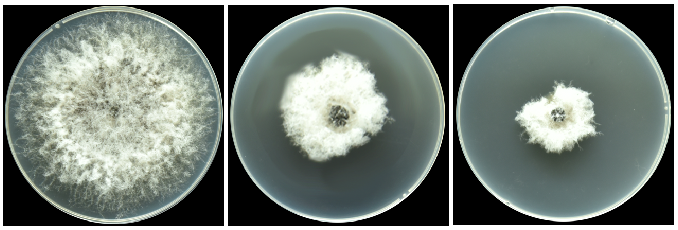

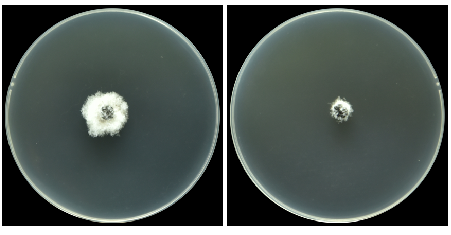


Difenoconazole


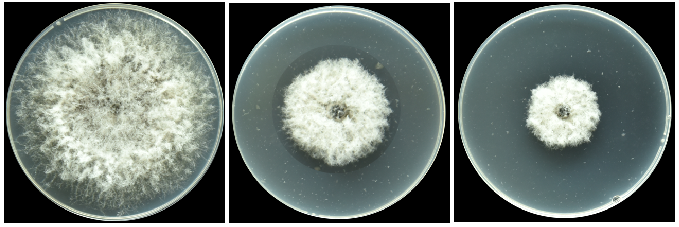


Azoxystrobin


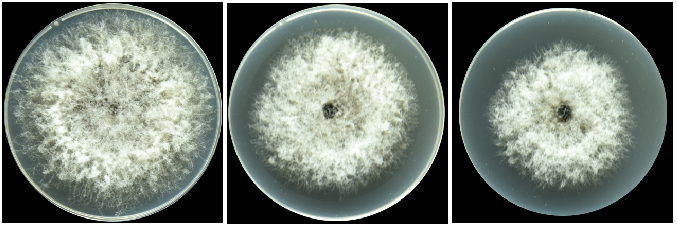


Myclobutanil


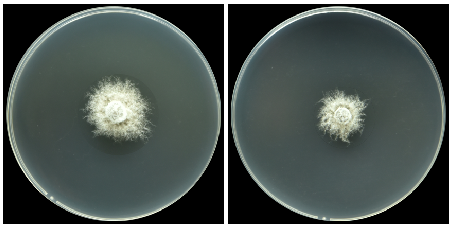

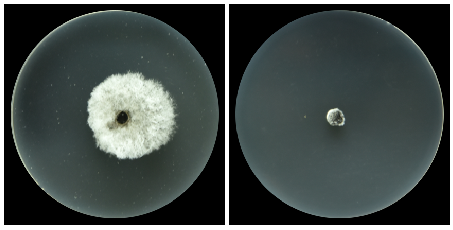


1. **CK 10^-5^ 10^-4^ 10^-3^ 10^-2^**

**E**


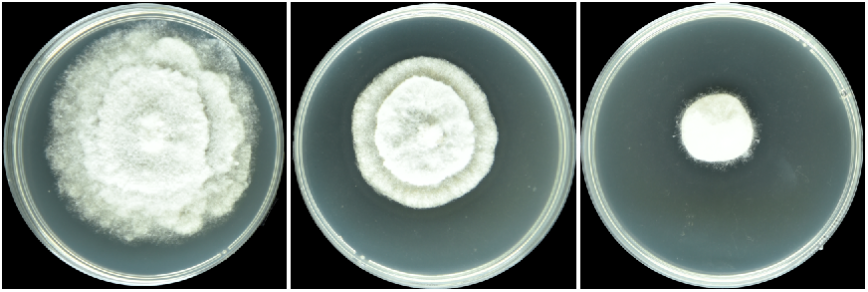

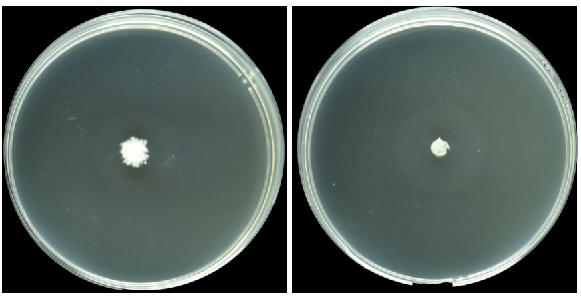


Pyraclostrobin


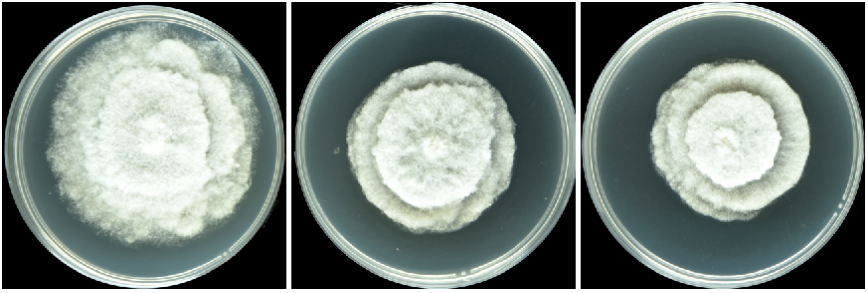

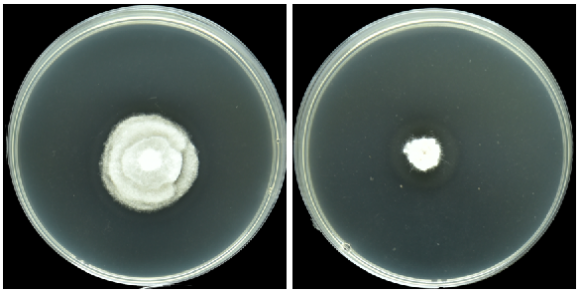


Prochloraz (copper salt)


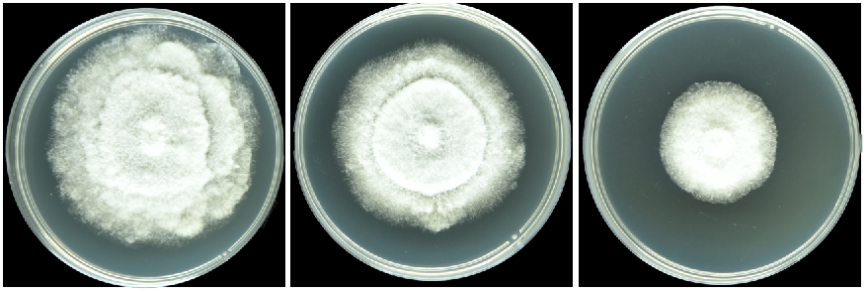


Azoxystrobin


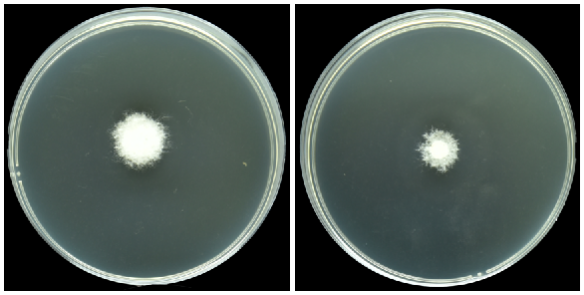

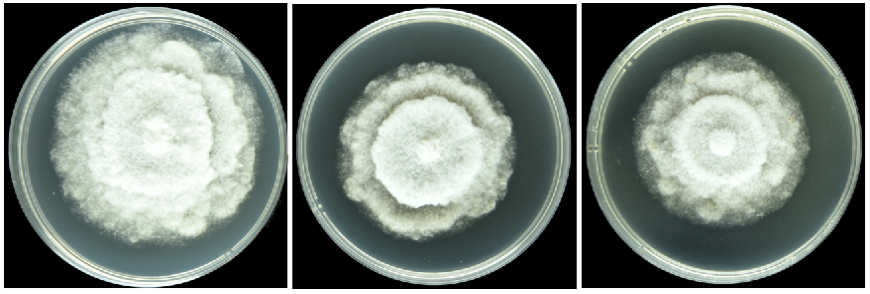

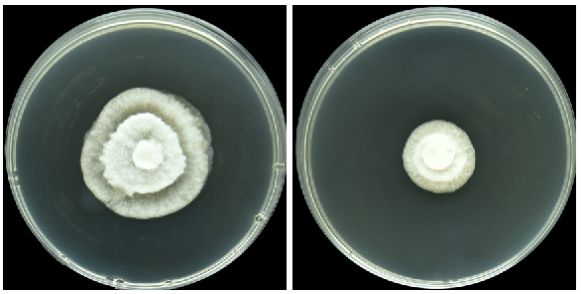


Chlorothalonil

1. **CK 10^-5^ 10^-4^ 10^-3^ 10^-2^**

**Figure S6.** Fungicides with obvious growth inhibition effects on pathogens E1 (A), F7 (B), L3 (C), N9 (D) and P4 (E).

**
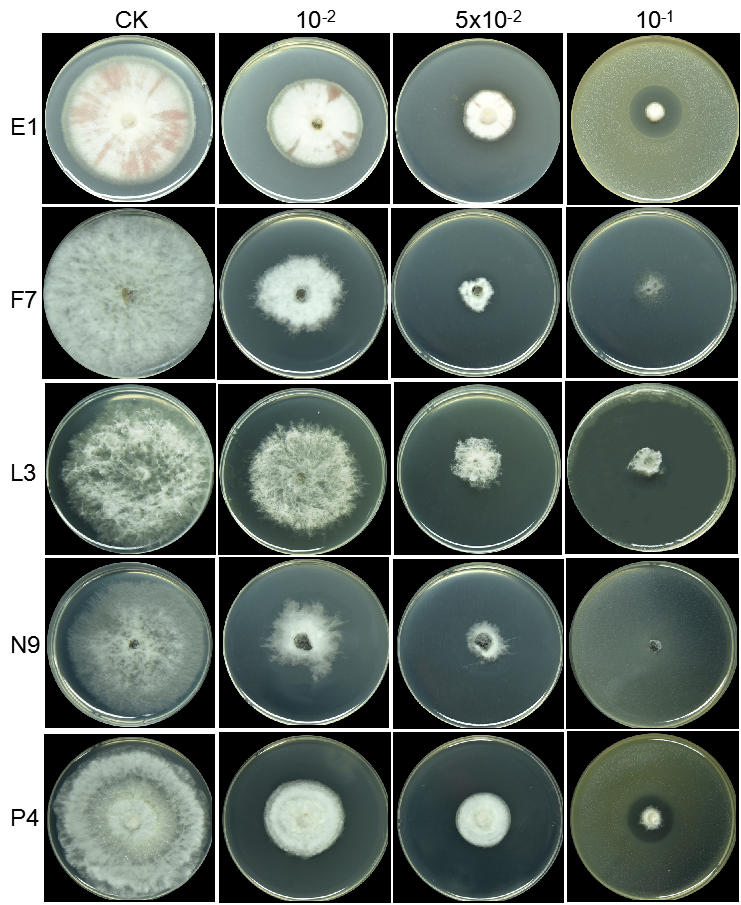
**

**Figure S7. Inhibition effects of zeamines from *Dickeya zeae* EC1 on pathogens E1, F7, L3, N9 and P4.** *D. zeae* EC1 (pathogen of rice foot rot disease) was cultured in LS5 medium, and 1, 5 and 10 mL of its supernatants (OD_600_ = 1.8) were respectively added into 100 mL PDA medium for growth inhibition assay. Mycelial plugs (5 mm in diameter) were punched out from the edge of a 5-day-old fungal colony and placed onto the centre of the toxic plates. Controls were set as 10% of ΔzmsA (*zmsA* gene deletion EC1 mutant which could not produce zeamines [13]) supernatant was added into the PDA medium. All plates were incubated at 28 °C until CK mycelia developed to the edge.
